# Sustainable Waste Tire Rubber Granule Concrete: Preparation, Mechanical Performance and Field Application for Pressure Relief in High-Ground-Stress Soft Rock Roadways

**DOI:** 10.3390/ma19091870

**Published:** 2026-05-01

**Authors:** Wei-Guo Qiao, Yun-Rui Zhao, Yue Wu, Wei-Min Cheng, Yin-Ge Zhu

**Affiliations:** 1 Shandong Provincial Key Laboratory of Civil Engineering Disaster Prevention and Mitigation, College of Civil Engineering and Architecture, Shandong University of Science and Technology, Qingdao 266590, China; skd991291@sdust.edu.cn (W.-G.Q.); 202383040077@sdust.edu.cn (Y.-R.Z.); zhuyg0221@sdust.edu.cn (Y.-G.Z.); 2 College of Safety and Environmental Engineering, Shandong University of Science and Technology, Qingdao 266590, China; wmcheng@sdust.edu.cn

**Keywords:** rubber granule concrete, waste tire recycling, mechanical properties, high-ground-stress soft rock roadway, floor heave control

## Abstract

**Highlights:**

**What are the main findings?**

- Develops and optimizes rubber granule concrete (12% content) as a stress-release layer.
- Validates field performance: ~64% stress reduction in high-ground-stress soft rock roadways.
- Achieves dual benefits of geotechnical stability and waste tire diversion.

**What are the implications of the main findings?**

- Proposes a high-volume, high-value pathway for waste tire valorization in geotechics.
- Provides a complete case study from material design to field deployment.
- Offers a sustainable solution for waste management and deep roadway stability.

**Abstract:**

Waste tire disposal and high-ground-stress soft rock roadway instability are pressing global challenges. This study develops sustainable rubber granule concrete (RGC) using waste tire rubber as a key component, aiming to realize waste valorization and floor heave control. RGC’s mechanical properties (uniaxial/triaxial compression, compressibility, ductility) were systematically tested, and its pressure relief mechanism was validated via finite element analysis (ABAQUS/FLAC) and 60-day field monitoring. Results show that RGC with optimal parameters (12% rubber content, 3–4 GPa elastic modulus, 250–350 mm thickness) achieves 64% bottom stress reduction and >40% displacement control. The material’s excellent energy absorption and flexibility address the brittleness of conventional concrete, ensuring stable support in high-stress environments. This work provides a sustainable, cost-effective concrete modification strategy, bridging waste recycling and geotechnical engineering, with broad implications for low-intensity, high-toughness material applications.

## 1. Introduction

The massive accumulation of waste tires has become a global environmental challenge, as conventional disposal methods (landfilling, incineration) cause ecological pollution and resource waste, while mainstream recycling mostly produces low-value downcycled products [1]. Simultaneously, deep underground engineering faces severe stability problems of floor heave under high geostress; conventional support methods (anchor-cable support, drilling pressure relief) fail to achieve effective deformation control due to the brittleness of ordinary concrete. Rubber granule concrete (RGC) has been proven to have excellent deformability and energy absorption capacity, but existing studies on RGC are mostly limited to laboratory mechanical property tests and numerical simulations, with scarce systematic research on its pressure relief mechanism and practical field application in high-ground-stress soft rock roadways [2]. In addition, the optimal parameter system of RGC for roadway floor heave control and its synergistic support design with conventional geotechnical support structures have not been clarified [3,4]. To bridge the above research gaps and realize the dual goals of waste tire valorization and deep roadway stability control, this study systematically investigates the mechanical properties of waste tire-derived RGC, optimizes its key parameters for pressure relief via theoretical derivation and numerical simulation, and verifies its engineering effectiveness through field application in a high-ground-stress soft rock roadway in Hebei. This research provides a sustainable material solution and engineering design reference for the integrated management of waste tires and the control of deep roadway floor heave.

Research on RGC has primarily involved laboratory tests and numerical simulations.

Laboratory tests reveal the mechanical and fatigue properties of rubber-bearing concrete. Bu [5] established a rubber-pumice concrete basis for green buildings. He et al. [6] enhanced rubber–cement interactions via modification. Some scholars [7,8] linked rubber content/form to reduced strength but improved durability and optimized proportions. Liu et al. [9] reported that compared with ordinary concrete, 5–15% rubber improved the fatigue life.

Similarly, Feng et al. [10] modeled the positive effects of polyvinyl alcohol (PVA)-reinforced rubber granular concrete. Li et al. [11] demonstrated its deformation resistance. Feng et al. [12] noted that concrete containing 100 mm-diameter rubber particles had reduced strength. Chen et al. [13] validated steel pipe-filled concrete via finite element method. A comparison of the relevant results provided a basis and support for engineering design. Liu et al. [14] correlated rubber content/size with finite element simulations.

Most studies have focused on single properties of rubber-bearing concrete, and multicharacteristic analysis is lacking. Kang et al. [15] enhanced cement–rubber interfaces via surface treatment, mitigating mechanical deterioration. Han et al. [16] linked rubber content/pretreatment to stress–strain behavior for design standards. Ahmad et al. [17] noted potential and challenges, requiring further research for strength optimization. Feng et al. [18] revealed strain rate effects and that 30% rubber reduced the brittleness of concrete. Zhang and Zhao [19] demonstrated the superior fatigue life of rubber granular concrete under inhibitory conditions. Collectively, these studies clarify rubber’s role in bending performance, brittleness, stress, and fatigue, supporting engineering applications.

While RGC has been widely studied, existing research neglects deep engineering in complex geological conditions, particularly its feasibility, environmental impact, and cost-effectiveness in high-stress roadways. Systematic investigations into such applications remain scarce.

Tunnel construction with low surrounding rock stress allows ordinary support to control floor deformation, but deep high-ground-stress roadways face severe bottom drum due to elevated stress, compromising safety. Conventional methods (drilling pressure release, anchor–cable support) stabilize sidewalls but inadequately control bottom drum [20]. This study reuses waste rubber in concrete, utilizing its stress-transfer properties in shotcrete to reduce bottom stress and deformation.

This reuse of waste tires reduces both environmental burden and engineering material costs. In particular, the application of RGC in the field of stability reinforcement of soft rock roadways under high stress has innovative importance.

Although existing studies have explored the energy absorption and stress relief properties of rubberized concrete, most of them focus on laboratory tests or numerical simulations, with no practical field application in underground roadway floor heave control [12,13]. This study bridges this research gap by integrating waste tire recycling with deep geotechnical engineering, and its fundamental novelties are: (1) Field application innovation: the first time to apply waste tire-derived RGC as a pressure-relief layer in actual high-ground-stress soft rock roadway floor heave control, with validated engineering effectiveness; (2) Parameter optimization innovation: optimizing RGC parameters (rubber content, thickness, modulus) for geotechnical stress relief via a combined method of laboratory tests-theoretical derivation-numerical simulation, forming a set of applicable design parameters; (3) Synergistic design innovation: proposing a hybrid support system of RGC pressure-relief layer + conventional anchor-cable support, which balances stress relief and structural stability for deep roadways.

Summary of Key Symbols and Abbreviations:

All abbreviations and symbols used in the manuscript are consistent throughout, with SI units applied for physical quantities. Key definitions are summarized as follows for quick reference:

Abbreviations: RGC (Rubber Granule Concrete, waste tire-derived concrete used as a pressure-relief layer); UCS (Uniaxial Compressive Strength, maximum compressive strength of RGC under uniaxial loading); ABAQUS 6.13/FLAC3D 6.00 (finite element software used for numerical simulation); ASTM (American Society for Testing and Materials, testing standard source); ISO (International Organization for Standardization, testing standard source).

Formula Symbols: effective span of the fixed-end rock beam *l* (m); vertical deflection of the rock beam at cross-section *x* as *ω* (m); horizontal geostress acting on the rock beam *P* (kN); vertical support force of the roadway rib on the rock beam *F* (kN); uniform vertical load on the rock beam (including self-weight) *q* (kN/m); flexural rigidity of the rock beam *EI*, (N·m^2^); horizontal position of the rock beam cross-section *x* (m); critical bending stress causing plastic deformation of the rock beam *σ*_cr_ (MPa); critical bending stress *P*_cr_ (MPa); C1 and C2 are undetermined coefficients.

## 2. Materials and Methods

The RGC adopted in this study comprises cement, water and rubber.

### 2.1. Materials

#### 2.1.1. Cement

Utilizing the specified materials, including C32.5 Portland cement (key chemical compositions: CaO 64.86%, SiO_2_ 20.31%, Al_2_O_3_ 5.94%, Fe_2_O_3_ 4.26%; loss on ignition 1.38%), C20 shotcrete was produced. The selection of this cement grade was based on its common application in deep underground shotcrete.

#### 2.1.2. Water

The water used in the experiments was obtained from the Shandong Provincial Key Laboratory of Disaster Prevention and Mitigation.

#### 2.1.3. Rubber

**Figure 1:**
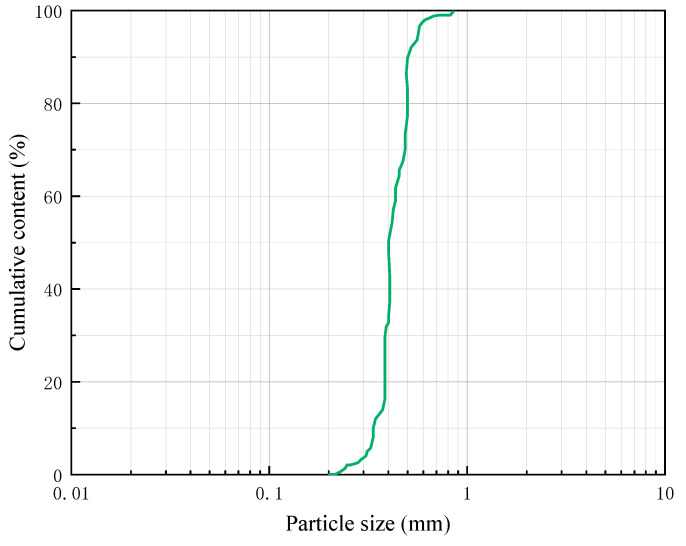
The particle gradation test of RGC.

The discarded rubber tires were collected, broken down, cleaned, and ground to produce rubber particles (key chemical compositions: rubber hydrocarbon 43.69%, carbon black 26.61%, isoprene 13.35%, acetone 12.99%; ash, fiber, and metal contents totaling <2%). The particle gradation of RGC is shown in Figure 1, with its maximum particle size being 0.85 mm.

### 2.2. Methods

#### 2.2.1. Laboratory Test

**Figure 2:**
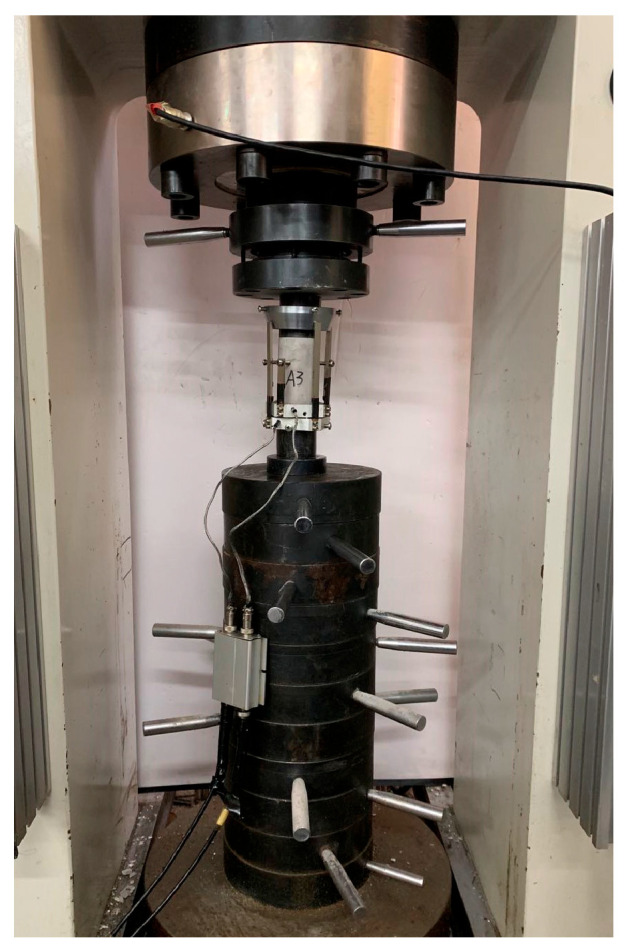
TAW-2000 electrohydraulic servo rock pressure tester.

All uniaxial and triaxial compression experiments in this study were conducted using a TAW-2000 electrohydraulic servo rock pressure tester, Changchun Rising Sun Testing Instruments Co., Ltd., Changchun, China (equipment schematic diagram is shown in Figure 2). The experimental scheme adopted C32.5 Portland cement as the cementitious material, with rubber particles replacing natural fine aggregate by mass fraction at gradients of 0% (control), 5%, 10%, 15%, 20%, 25%, 30%, and 40%. All mixtures used a fixed water-cement ratio of 0.57 and uniformly graded fine aggregate with a particle size of 0.85 mm (no admixtures were added), and the prepared RGC mixtures were cast into standard specimens with dimensions of φ150 mm × 300 mm.

The 0–40% rubber content gradient was selected based on two considerations: (1) literature basis: existing studies on rubberized concrete for geotechnical stress relief have tested 0–50% rubber content to explore the correlation between rubber content and deformability/energy absorption [7,8], and (2) pre-experiment results: our pre-tests showed that rubber content < 40% can ensure basic workability of RGC shotcrete (slump ≥ 120 mm) for underground construction, while >40% leads to severe segregation and unworkability. A 10% interval was set to accurately capture the mechanical property variation trend with increasing rubber content.

The specimen size of φ150 mm × 300 mm was selected in accordance with ASTM C39/C39M-21 and ISO (International Organization for Standardization, testing standard source) 4014:2021, which is the standard cylindrical specimen size for concrete mechanical property tests and ensures the representativeness of the test results (the diameter-height ratio of 1:2 avoids the size effect of compressive strength tests). The rubber content gradient of 0–40% (by mass fraction) was determined based on two considerations: (1) international standard and research basis: existing studies on RGC for geotechnical engineering (ASTM-recommended) have adopted 0–50% rubber content to explore the mechanical property variation trend; (2) engineering practicality: pre-tests show that rubber content < 40% can ensure the workability of RGC shotcrete for underground engineering (slump ≥ 120 mm), while content >40% causes severe segregation and cannot be applied in field construction. A 10% interval was set to accurately capture the quantitative relationship between rubber content and RGC’s pressure relief performance.

RGC was prepared via a forced-action mixer: materials were weighed to the designed ratio, mixed for 100 s, poured into molds, and compacted on a vibration table (per GB/T 50080-2016 [21]). Formed samples were cured indoors at 20 ± 5 °C for 24 h, demolded, labeled, and transferred to a standard curing chamber (20 ± 2 °C, RH > 95%). Compressive strength was tested using a computer-controlled electronic universal testing machine. Test equipment (pressure testing machines, vibration tables) was regularly calibrated by qualified metrology units with records retained.

Test procedure: Sample surfaces were smoothed with an angle grinder, grouped, and positioned in the testing machine. Parameters were configured, the pressure platform was lowered, and real-time stress curves were recorded. The machine stopped at ultimate compressive strength, and results were saved for subsequent batches.

Freeze-thaw cycle tests used a high-low temperature alternating wet-dry heat cycle box (temperature: −40 °C~150 °C, humidity: 20~98%, heating/cooling rate: 1~3 °C/min). Temperature and humidity were program-set, and cycles were completed automatically. Mechanical parameters were tested post-cycles via the aforementioned methods.

An extension meter was used to obtain the displacement variation. (1) First, connect the extensometer to the computer host, and then set the control test equipment software to the “extensometer usage” mode to acquire the relevant measurement data. (2) The spacer was clamped between the lever arm and the gauge rod, and the two lever arms were pressed so that the two blades directly touched the sample. (3) The extensometer was secured to the sample using a spring or rubber band. After assembly, the spacer was removed from the gauge rod, and a gap of approximately 0.5 mm was maintained between the force arm and the gauge rod. The purpose of securing with a spring or rubber band is to prevent blade slippage. When the sample is fixed, a slight groove can also be made on it to secure the blade. (4) Zero adjustment of the extensometer. (5) Conduct experiments. (6) When the material deformation reaches the yield limit, the extensometer should be removed quickly; otherwise, the sample may be damaged when it breaks.

All RGC laboratory specimens (φ150 mm × 300 mm) were prepared in accordance with the Chinese national standard for concrete mechanical tests (GB/T 50081-2019 [22]). To evaluate the scale effect of RGC mechanical properties, a numerical scale-up analysis was conducted, where the mechanical behavior (stress-strain curve, energy absorption capacity) of RGC specimens with sizes from laboratory scale (φ150 mm × 300 mm) to field full scale (3 m × 3 m × 0.3 m) was simulated. The results show that the key mechanical properties of RGC change by <8% with the increase of specimen size, indicating that the scale effect has a negligible impact on the engineering application of RGC.

Compressive strength tests were conducted in accordance with ASTM C39/C39M-21 [23] (Standard Test Method for Compressive Strength of Cylindrical Concrete Specimens) and ISO 4014:2021 [24] (Concrete—Sampling and testing of fresh concrete). Freeze-thaw cycle tests were performed following ASTM C666/C666M-23 [25] (Standard Test Method for Resistance of Concrete to Rapid Freezing and Thawing). Triaxial compression tests were carried out in accordance with ISO 17892-8:2020 [26] (Geotechnical investigation and testing—Laboratory testing of rock—Part 8: Triaxial compression tests).

#### 2.2.2. Theoretical Derivation

Theoretical derivation: The deep roadway bottom was simplified as a fixed-end rock beam, and its mechanical characteristics were analyzed to support subsequent research.

#### 2.2.3. Numerical Simulation

Numerical Simulation: The finite element software ABAQUS 6.13 was utilized to conduct simulations under various conditions and parameters. Subsequently, the software FLAC 6.0 was applied to analyze these simulation outcomes and determine the optimal support scheme.

#### 2.2.4. Engineering Applications

Engineering application: The optimal support scheme was applied to a deep roadway site, with 60-day deformation monitoring to verify results.

## 3. Results of Mechanical Characteristics of RGC

This section analyzes RGC’s mechanical characteristics under uniaxial and triaxial stress states (theoretical deduction included). Uniaxial/triaxial experimental methods follow previous studies [27] and are not repeated.

Rubber particle contents (mass ratio): 0% (I), 5% (A), 10% (B), 15% (C), 20% (D), 25% (E), 30% (F), 40% (H).

### 3.1. Mechanical Properties of Single-Axis Loading

**Figure 3:**
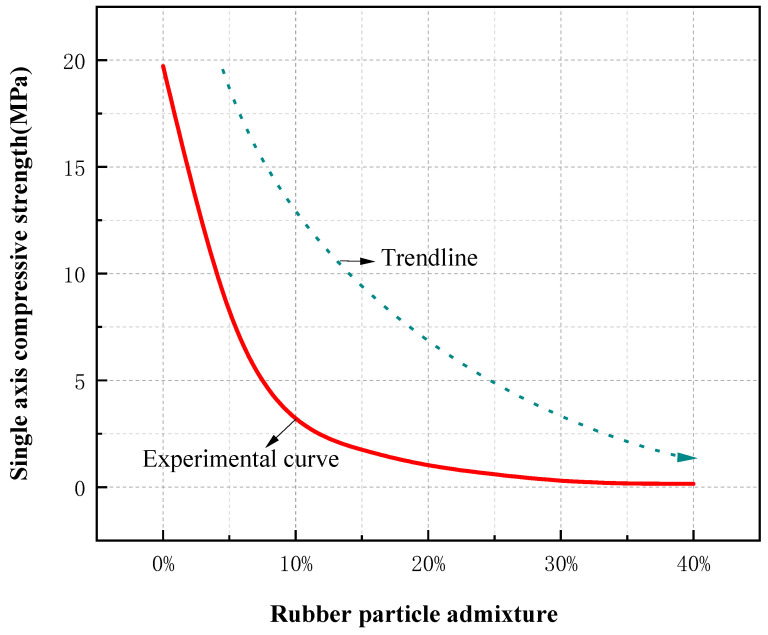
UCS (uniaxial compressive strength) of RGC with different rubber particle contents (the blue dotted line shows the trend).

**Figure 4:**
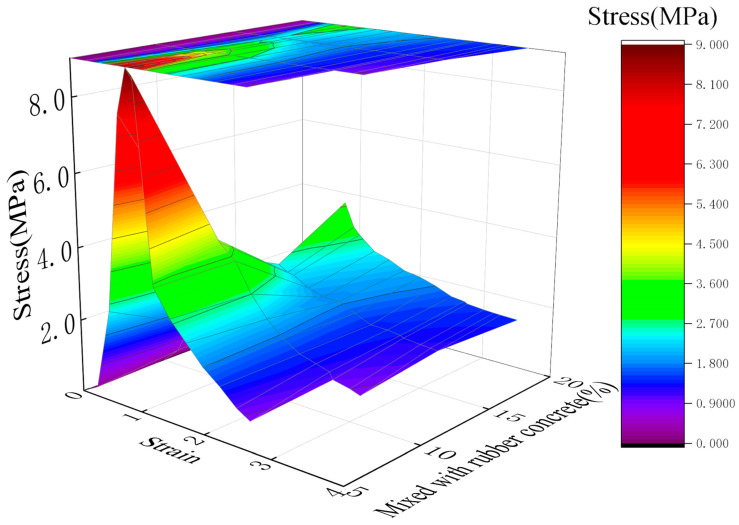
Stress–strain curves of concrete with different proportions of rubber particles (5–20%).

The uniaxial compression mechanical properties of RGC with rubber content gradients of 0–40% were tested in accordance with ASTM C39/C39M-21, and the key results are as follows:

The uniaxial compressive strength (UCS) of RGC decreased gradually with the increase of rubber content (Figure 3), from 32.5 MPa (0% rubber content) to 8.2 MPa (40% rubber content). The elastic modulus of RGC also showed a decreasing trend (Figure 4), with the slope of the linear elastic segment of the stress-strain curve reduced by 68% when the rubber content increased from 0% to 40%.

**Figure 5:**
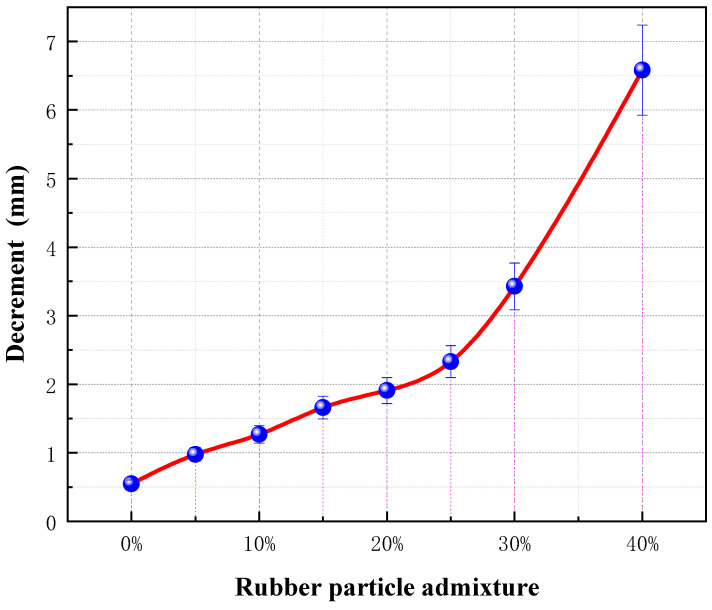
Compressibility of RGC with different proportions of rubber particles.

**Figure 6:**
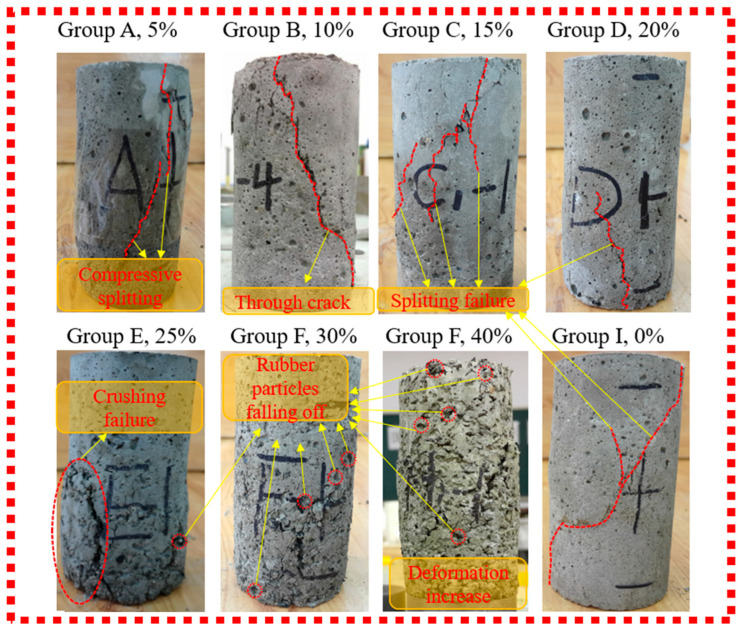
Uniaxial failure of RGC samples.

**Figure 7:**
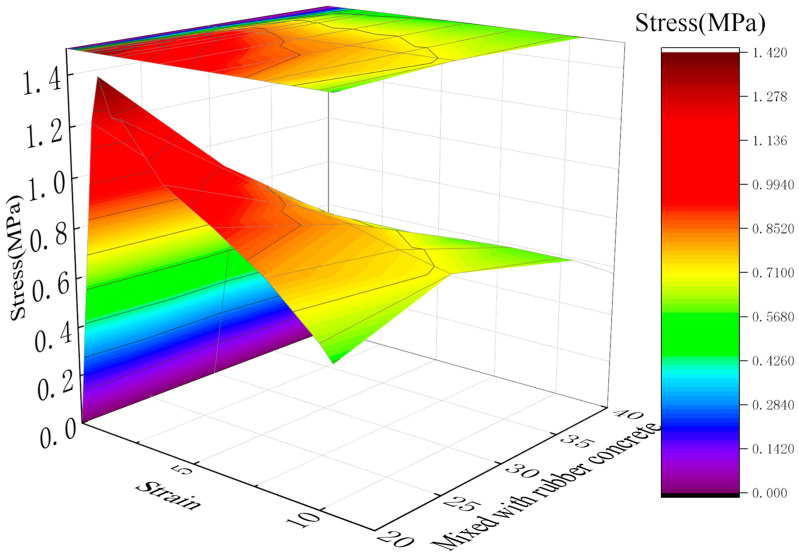
Stress–strain curves of concrete with different proportions of rubber particles (20–40%).

In contrast, the compressibility of RGC increased significantly with the increase of rubber content (Figure 5 and Figure 6), from 1.25 mm (0% rubber content) to 6.59 mm (40% rubber content), with a sharp increase when the rubber content exceeded 30%. The peak strain of RGC increased by 52% when the rubber content was 10–15% (Figure 7), indicating that the appropriate rubber content can significantly improve the ductility of concrete.

This variation trend is mainly attributed to the microstructural characteristics of RGC: rubber particles have low strength and poor interfacial bonding with the cement matrix, leading to the formation of weak interfaces that reduce UCS and elastic modulus; meanwhile, the viscoelasticity of rubber particles enables large deformation under load, thereby enhancing the compressibility and ductility of RGC. The decrease of UCS and elastic modulus of RGC with the increase of rubber content is mainly due to the following two reasons: (1) the rubber particles have low strength and poor bonding performance with the cement stone matrix, leading to the formation of weak interfaces in RGC, which are the first to generate microcracks under load; (2) the rubber particles are elastic materials with low elastic modulus, which reduce the overall stiffness of RGC when incorporated into concrete. The increase of RGC’s compressibility and ductility is attributed to the viscoelasticity of rubber particles, which can undergo large elastic deformation under load and absorb the strain energy generated by concrete deformation, thus delaying the occurrence of brittle failure of concrete.

### 3.2. Mechanical Properties in the Triaxial State

**Figure 8:**
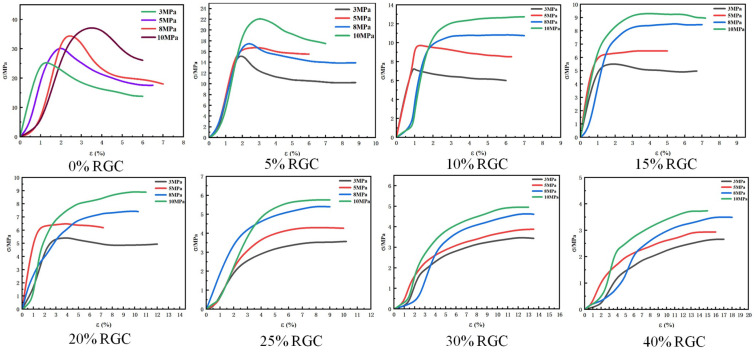
Stress-strain curves of RGC samples under different conditions (triaxial tests).

Figure 8 presents the stress-strain curves of RGC obtained under different confining pressures (3, 5, 8, and 10 MPa). An analysis of Figure 8 is provided below: For a fixed rubber content, an increase in confining pressure leads to a shift in the peak compressive strength to a higher strain level, accompanied by an increase in both the ultimate strain and the compressive strength of the specimen.

**Figure 9:**
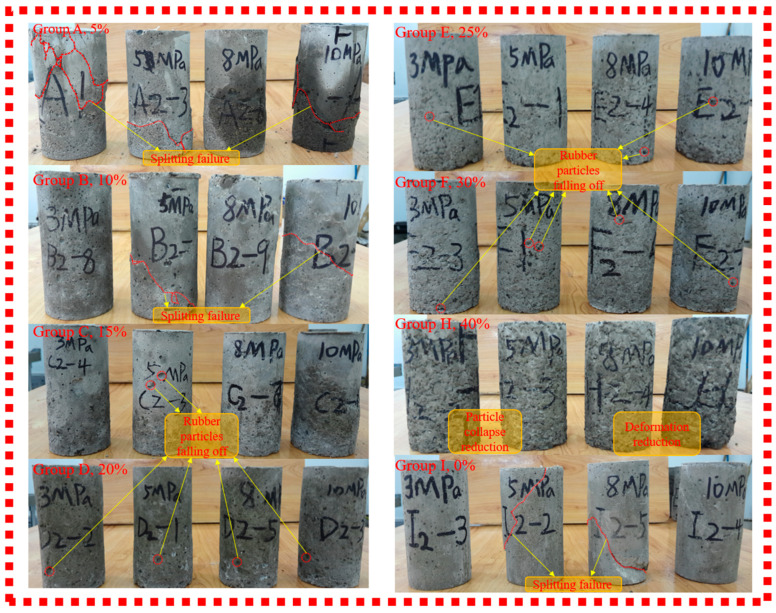
Destruction morphology of RGC samples under triaxial conditions.

The failure characteristics of the samples (triaxial tests) are illustrated in Figure 9, which includes groups with rubber contents of 5% (A), 10% (B), 15% (C), 20% (D), 25% (E), 30% (F), 40% (H), and plain concrete (I).

Figure 9 shows: <5% rubber (Groups A, I)—Group I: cleavage deformation, Group A: surface spalling; >10% rubber—middle bulging, no obvious damage, good integrity; compressibility increases with rubber content. Further analysis determined that the optimal rubber content is 12%, with performance: slump 100 mm, apparent density 2140 kg/m^3^, elastic modulus 2.64 GPa, flexural strength 0.81 MPa.

The rubber content was optimized to a range of 10–15% for RGC applied in high-ground-stress roadway support, which achieves a ≥30% increase in tensile strain capacity (enhancing deformability and stress release) while maintaining a uniaxial compressive strength (UCS) of ≥20 MPa. This UCS value meets the minimum mechanical requirement for roadway shotcrete support in Chinese geotechnical engineering specifications, balancing deformability and structural bearing capacity.

### 3.3. Analysis of Variance

Variance analysis validated experimental results: 10% rubber content (5 uniaxial compression tests: 3.31, 3.15, 3.06, 3.36, 3.22 MPa) yielded variance 0.01455 MPa^2^, standard deviation 0.121 MPa, coefficient of variation 3.76% (<5%, indicating low dispersion and good repeatability). Remaining data were analyzed similarly, with experiments repeated for excessive errors. All mechanical property tests of RGC were conducted on 5 parallel specimens for each rubber content gradient, in accordance with the Chinese national standard GB/T 50081-2019 [22]. The test results are expressed as mean value ± standard deviation (SD), and the coefficient of variation (CV) was calculated to evaluate data dispersion. For all mixtures, the CV of UCS, elastic modulus and strain capacity was <9%, which is within the acceptable range for concrete mechanical tests [9], indicating good repeatability and reliability of the experimental results.

### 3.4. Analysis of the Mechanical Characteristics of the Roadway Bottom

**Figure 10:**
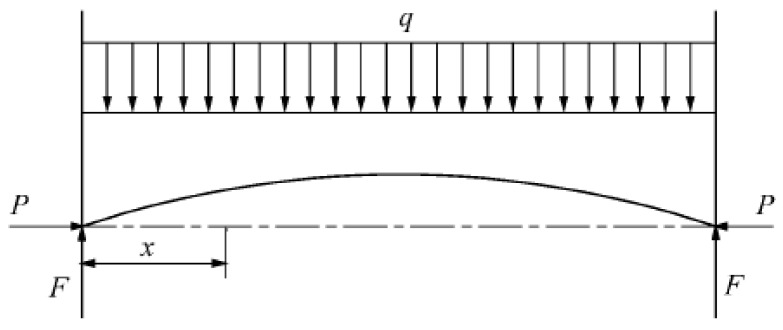
Stress model of the rock beam at the roadway bottom.

High ground stress induces bending deformation of the rock stratum, and the roadway floor is simplified as a fixed-end rock beam (Figure 10). The key parameters and their notations are defined as follows: effective span of the fixed-end rock beam *l* (m); vertical deflection of the rock beam at cross-section *x* as *ω* (m); horizontal geostress acting on the rock beam *P* (kN); vertical support force of the roadway rib on the rock beam *F* (kN); uniform vertical load on the rock beam (including self-weight) *q* (kN/m); flexural rigidity of the rock beam *EI*, (N·m^2^); horizontal position of the rock beam cross-section *x* (m); critical bending stress causing plastic deformation of the rock beam *σ*_cr_ (MPa); critical bending stress *P*_cr_ (MPa).

The bending moment of the slab beam at any interface *x* is as follows:

(1) $$M(x)=P\omega -\frac{1}{2}qlx+\frac{1}{2}qx^{2}$$

The differential equation for the deflection of the bottom plate is as follows:

(2) $$El\omega^{\prime \prime }=-(P\omega -\frac{1}{2}qlx+\frac{1}{2}qx^{2})$$

where *EI* is the flexural strength of the rock beam.

Taking $k^{2}=\frac{P}{EI^{’}}$, Equation (2) can be simplified:

(3) $$\omega^{\prime \prime }+k^{2}\omega =\frac{qk^{2}}{2p}(lx-x^{2})$$

Equation (3) has the following general solution:

(4) $$\omega =C_{1}\sin kx+C_{2}\cos kx-\frac{q}{2p}x^{2}+\frac{ql}{2P}x+\frac{q}{k^{2}p}$$

where *C*_1_ and *C*_2_ are undetermined coefficients.

According to the boundary conditions $\omega_{x=0}=0$ and $\omega_{x=\frac{1}{2}}^{’}=0$, the following can be obtained:

$$C_{1}=-\frac{q}{k^{2}p}\tan\frac{kl}{2}\;C_{2}=-\frac{q}{k^{2}p}$$

$\omega_{x=l}=0$. If *x* = *l* and *C*_1_ and *C*_2_ in Equation (4) are combined, the following equation is obtained:

(5) $$\omega =-2\frac{q}{k^{2}p}\sin\frac{kl}{2}(1-\sin\frac{kl}{2})$$

In Equation (5), letting ω=0 and $\sin\frac{kl}{2}(1-\sin\frac{kl}{2})=0$, we obtain $\frac{kl}{2}=\frac{n\pi }{2}$ (*n* = 0, 1, 2, 3, …); from this result, $k=\frac{n\pi }{l}$, which can be rewritten as $k^{2}=\frac{P}{EI^{’}}$, yielding the following:

(6) $$P_{\mathrm{cr}}=\frac{nEI\pi^{2}}{l^{2}}\;(n=0,1,2,3,\ldots )$$

The value of *n* can be 0, 1, 2, 3 or any other integer. However, at these pressures, only the minimum pressure that destabilizes the rock beam at minimal bending is the critical force needed. In this case, a value of *n* = 0 is nonsensical. Therefore, *n* = 1 yields the following:

(7) $$P_{\mathrm{cr}}=\frac{EI\pi^{2}}{l^{2}}$$

When the rock beam is subjected to the critical stress and cannot maintain a stable balance, its cross-sectional area is *A*. Then, the critical stress on the cross section is:

(8) $$\sigma_{\mathrm{cr}}=\frac{P_{\mathrm{cr}}}{A}$$

**Figure 11:**
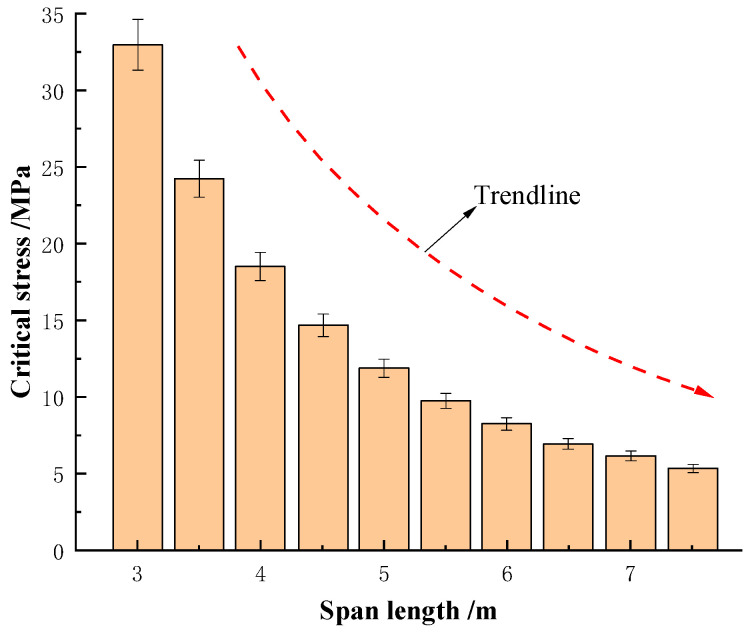
Relationship between the roadway width and critical stress.

Suppose that the bottom layer of a roadway is 0.5 m thick and 1 m wide. The flexural rigidity of this rock layer is 15 × 10^6^ N·m^2^. Figure 11 summarizes the relationship between roadway width and the critical stress.

Figure 11 shows roadway width significantly affects the bottom plate’s critical deformation load: 3 m → 6 m width reduces critical load from 34 MPa to 9 MPa (75% decrease), increasing deformation likelihood [20,28,29]. These results support subsequent support scheme design.

## 4. Numerical Simulation Study of Pressure Release Support for RGC

The software ABAQUS was used to discuss the action mechanism of rubber particles in roadways according to different schemes.

### 4.1. Model Building

#### 4.1.1. Development of the Numerical Simulation Model

Roadway excavation disturbs surrounding rock [30,31,32]. To reduce boundary effects, the model size (33 m × 33 m × 20 m) is 10 times the roadway width (simulation size >3× diameter: ~5% error; >5× diameter: ~1% error). This model is a first-order approximation and does not capture joints or anisotropy.

The proposed numerical model is fully parameterized, where key inputs such as rock mass properties, in-situ stress field, and RGC material parameters can be easily adjusted. This flexibility enables the model to be adapted for preliminary performance predictions in other underground projects by simply inputting site-specific rock mechanics data.

**Figure 12:**
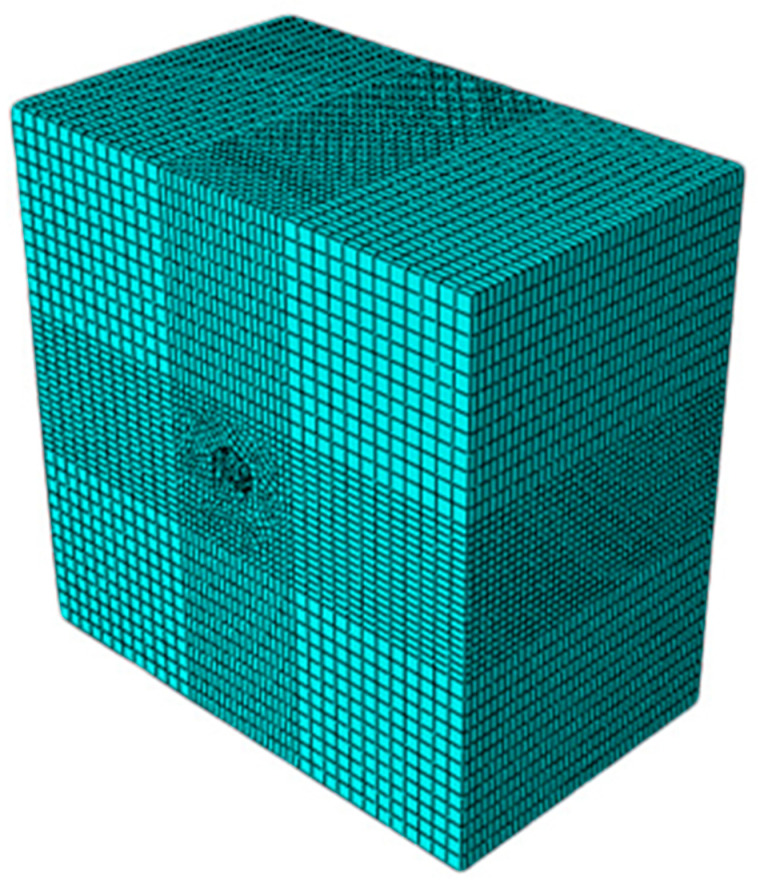
Schematic illustration of the high-stress soft-rock roadway numerical model.

Geological conditions: siltstone/mudstone [33]; boundary conditions: upper boundary vertical stress 21.52 MPa (800 m rock weight), lateral pressure coefficient 1.3. Mesh: hexahedral elements (aspect ratio ≤ 3, internal angles ≥ 45°), dense within 3× arch top diameter, gradual transition; constitutive model: Coulomb–Mohr criterion; structural damping factor: 0.03. The calculation model is shown in Figure 12.

#### 4.1.2. Determination of Surrounding Rock Parameters and Design of Support Schemes

Deep engineering surrounding rock stress: approximately 25 MPa (up to 30 MPa), UCS < 10 MPa. The surrounding rock (siltstone, sandy mudstone, mudstone) had the following mechanical parameters: siltstone (*E* = 13.0 × 10^3^ MPa, *μ* = 0.25, density = 26.9 kN/m^3^, cohesion = 9.8 MPa, internal friction angle = 37°); sandy mudstone (*E* = 4.5 × 10^3^ MPa, *μ* = 0.29, density = 26.1 kN/m^3^, cohesion = 1.9 MPa, internal friction angle = 30°; mudstone (*E* = 6.2 × 10^3^ MPa, *μ* = 0.26, density = 25.9 kN/m^3^, cohesion = 4.5 MPa).

The support structure parameters are as follows:

1. RGC layer: 400 mm thick.
2. Ordinary concrete layer: 400 mm thick.
3. Anchor rod: diameter Φ22 mm and a length of 2400 mm. Each rod had an anchoring length of 1000 mm, providing an ultimate anchoring force of 150 kN and an initial pre-tension force of 50 kN. The anchors were arranged with a row spacing of 800 mm and a spacing within the row of 500 mm.

The support schemes are as follows:

Scheme 1: 500 mm ordinary concrete (poured in two phases: 400 mm + 100 mm) with anchor bolts.

Scheme 2: 400 mm RGC + 100 mm ordinary concrete, followed by anchor bolts.

A simulation analysis was performed to compare the effectiveness of the two support schemes in high-stress soft rock roadways.

The surrounding rock mechanical parameters (siltstone, sandy mudstone, mudstone) were determined based on laboratory triaxial compression tests of in-situ rock samples (in accordance with ISO 17892-8:2020 [26]), which accurately reflect the actual geological conditions of the test site. The RGC material parameters (elastic modulus, UCS, Poisson’s ratio) were obtained from the laboratory test results of this study, ensuring the consistency between the numerical model and the actual material properties. The support structure parameters (anchor rod diameter, length, spacing, concrete layer thickness) were designed in accordance with the Chinese National Standard GB 50086-2015 [33] (Code for Design of Anchor Engineering) and the actual engineering experience of high-ground-stress soft rock roadways in China. The geostress parameters (vertical stress 21.52 MPa, lateral pressure coefficient 1.3) were derived from in-situ stress measurement results of the test site, ensuring the authenticity of the numerical model’s load conditions. All model parameters were verified by existing geotechnical engineering research [20,34] to ensure their rationality and applicability.

#### 4.1.3. Model Parameter Calibration: Connection Between Laboratory Tests and Numerical Simulation

All key material parameters of RGC in the ABAQUS/FLAC numerical model were directly determined and calibrated by our laboratory test results, to ensure the model’s authenticity and predictive ability: (1) The elastic modulus, UCS, Poisson’s ratio and stress-strain curve of RGC with different rubber contents were imported into the model as the constitutive parameters of the roadway bottom pressure-relief layer, replacing generic concrete parameters; (2) The rock mass parameters (siltstone/mudstone) were calibrated by laboratory triaxial compression tests of in-situ rock samples; (3) The theoretical derivation of the fixed-end rock beam model provided the critical load and boundary conditions for the numerical simulation (e.g., critical deformation load of roadway bottom plate), which was used as the initial loading condition of the model. In summary, the theoretical derivation defined the model’s mechanical boundary, while laboratory tests provided the accurate material constitutive parameters, forming a closed research loop of “theoretical guidance—experimental calibration—numerical simulation verification”.

### 4.2. Analysis of the Influence of RGC on the Pressure Release Effect

A comparative analysis was conducted on the stress, displacement, and support load in the surrounding rock for cases with and without the RGC layer.

#### 4.2.1. Numerical Simulation of the Concrete Layer Without Rubber Particles

A numerical simulation of the scenario with conventional concrete (without rubber) was carried out, with the key results summarized as follows.

1. Stress of surrounding rock

The bottom rock stress without a RGC layer stabilized at 14.1~16.0 MPa after anchor rod installation, with the lowest stress (14.07 MPa) at the middle bottom and symmetric stresses (16 MPa) at the left/right bottom corners.

**Figure 13:**
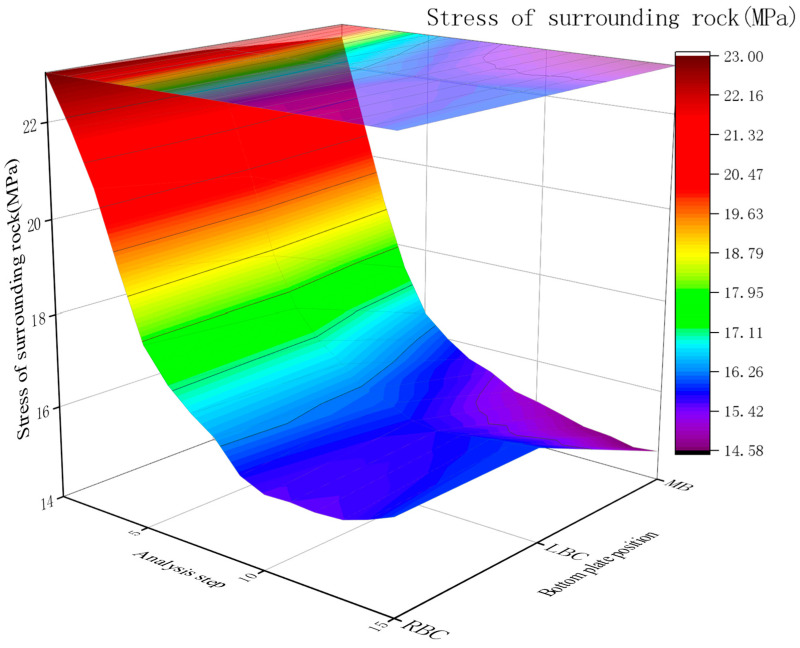
The stress curve of the surrounding rock without RGC layer.

The stress variation curve of the bottom rock layer under the condition without RGC support is shown in Figure 13.

Initial stress correlates with surrounding rock depth (high symmetry pre-excavation). Post-excavation, shear expansion in soft strata under high horizontal stress concentrates elevated stress around the roadway and base. Stress shifts significantly during excavation and concrete support; anchors initially have minimal effect (excavation-induced rock movement releases stress) but cause slight stress increase by restricting further deformation.

2. Displacement of surrounding rock

**Figure 14:**
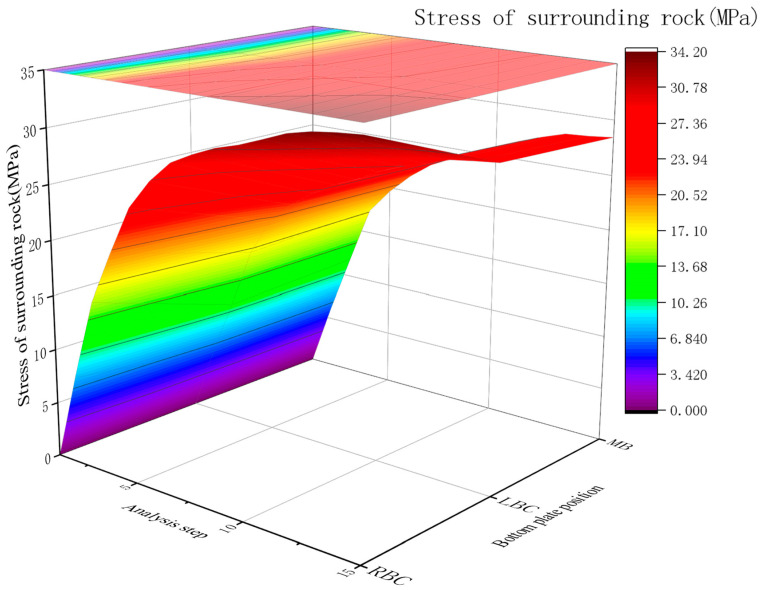
Displacement curve of the rock surrounding the roadway when there is no RGC layer.

The surrounding rock displacement without a RGC layer peaked at the middle bottom plate (34.1 mm), with symmetric displacements (about 29 mm) at the left/right bottom corners after anchor rod installation. Figure 14 illustrates the displacement curves at different locations of the bottom plate for the case without RGC support.

Post-excavation, surrounding rock (especially the bottom plate) exhibits large displacements due to high ground stress in deep soft rock roadways. Displacement changes significantly in the initial excavation stage, decreasing after concrete and anchor rod installation (consistent with basement rock layer stress changes, attributed to surrounding rock deformation and partial stress release).

3. Force acting on the roadway support structure

Without RGC support, the ordinary concrete layer had a stress of 58.23~66.54 MPa and the anchor rods had a stress of 79.22~90.78 MPa, both showing lower stress in the middle and higher stress on both sides. Initial displacement and concrete support deformation are significant, but bottom plate movement diminishes after concrete/anchor rod reinforcement (consistent with reduced basement rock stress from surrounding rock deformation and partial stress release).

#### 4.2.2. Numerical Simulation of RGC Between Surrounding Rock and Rigid Concrete

Analysis of RGC in High-Stress Soft Rock Roadways:

Without rubber granular concrete, the surrounding rock stress and support loads (rigid concrete/bolts) remain high. Numerical simulations of rubber granular concrete between rock and rigid concrete reveal the following:

1. Stress reduction:

Post application, the stress drops sharply during the rubber/ordinary concrete pouring stages (∼50% total reduction).

**Figure 15:**
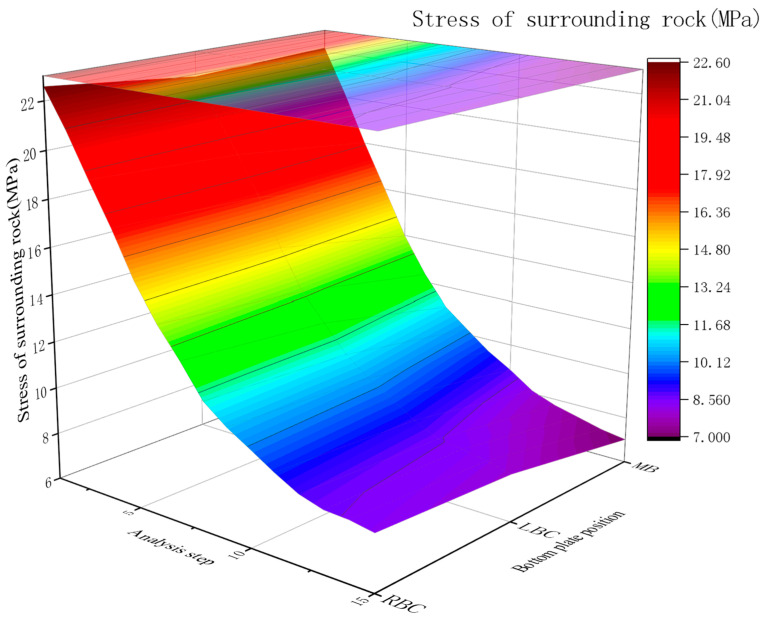
Stress curves of the RGC layer between the surrounding rock and ordinary concrete layer.

The addition of the RGC layer achieves a significant pressure relief effect on the roadway floor, with the total stress release rate reaching 65% (the middle bottom of the roadway has the best pressure relief effect). This key feature indicates that the RGC layer can effectively transfer and release the high ground stress of the surrounding rock, avoiding the stress concentration at the roadway floor, which is the core reason for the reduction of floor heave deformation and support structure load (Figure 15).

2. Displacement control

With a RGC layer, the surrounding rock displacement stabilized at 39.00~43.2 mm after anchor rod installation, with the maximum displacement (43.2 mm) at the middle bottom plate and a displacement release rate of 40%. The surrounding rock displacement increased from 19.5~23.00 mm post-excavation to 33.25~39.00 mm after adding the rubber layer.

**Figure 16:**
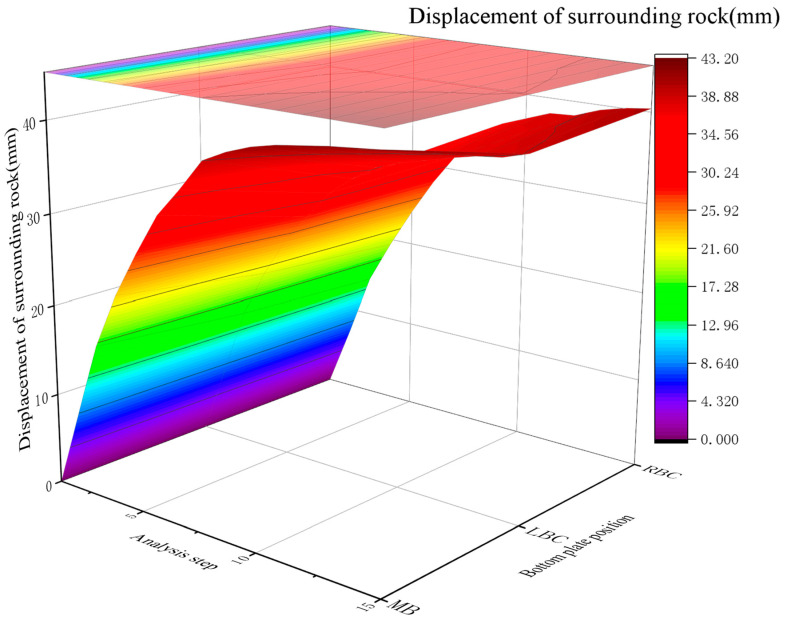
Displacement curve of the rock surrounding the roadway when a RGC layer is applied between the surrounding rock and the ordinary concrete layer.

Major displacements occurred during the excavation and concrete pouring stages (50% of the total displacement in the RGC placement phase), which is correlated with stress reduction (Figure 16).

3. Support loads:

The stress values of the support structure with a RGC layer were: RGC layer (18.97~20.32 MPa), ordinary concrete layer (68.56~70.67 MPa), and anchor rods (38.08~40.87 MPa). Compared with the scheme without rubber, from an average of 69.54 MPa to 39.69 MPa, with a more uniform stress distribution.

The results demonstrate that the RGC layer effectively releases ground stress, mitigates deformation, and reduces the load on the support structure, thereby validating its feasibility for application in high-stress soft rock roadways.

4. Analysis of the Pressure Release

**Figure 17:**
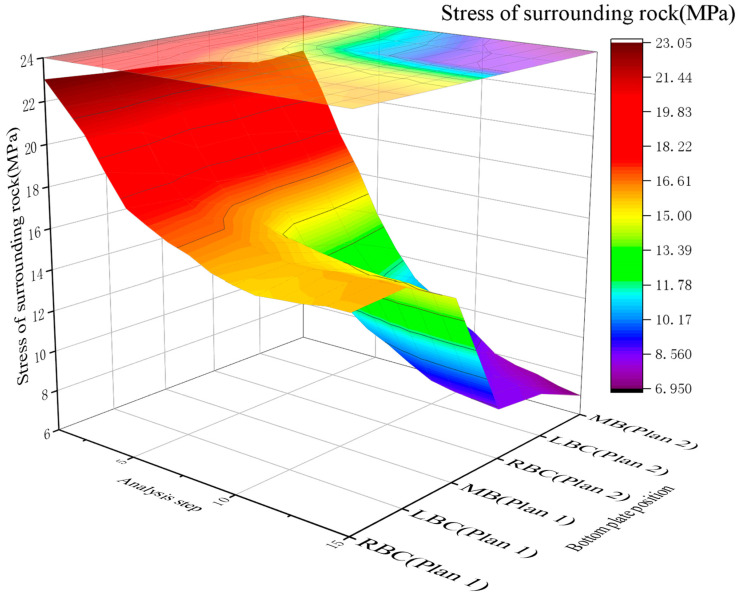
Comparison of the stresses in the surrounding rock in the two scheme.

**Figure 18:**
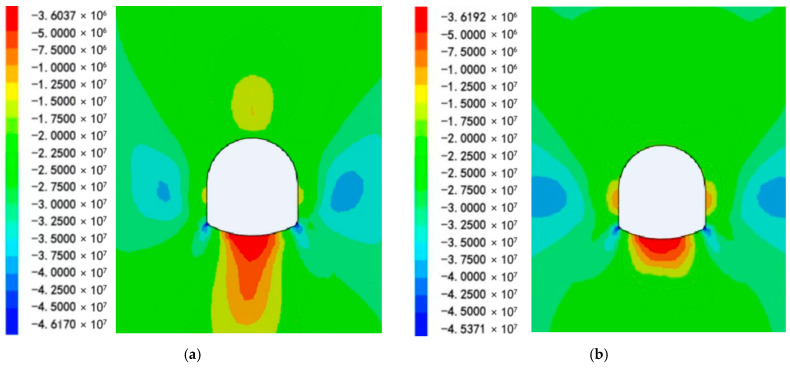
Roadway bottom rock stress map: (**a**) Roadway bottom rock stress map of Scheme 1; (**b**) Roadway bottom rock stress map of Scheme 2.

Figure 17 shows a comparative analysis of the surrounding rock stress values of Schemes 1 and 2. A stress cloud map of the two support schemes is shown in Figure 18a,b. The stress cloud maps of Schemes 1 and 2 are shown in Panels a and b, respectively.

**Figure 19:**
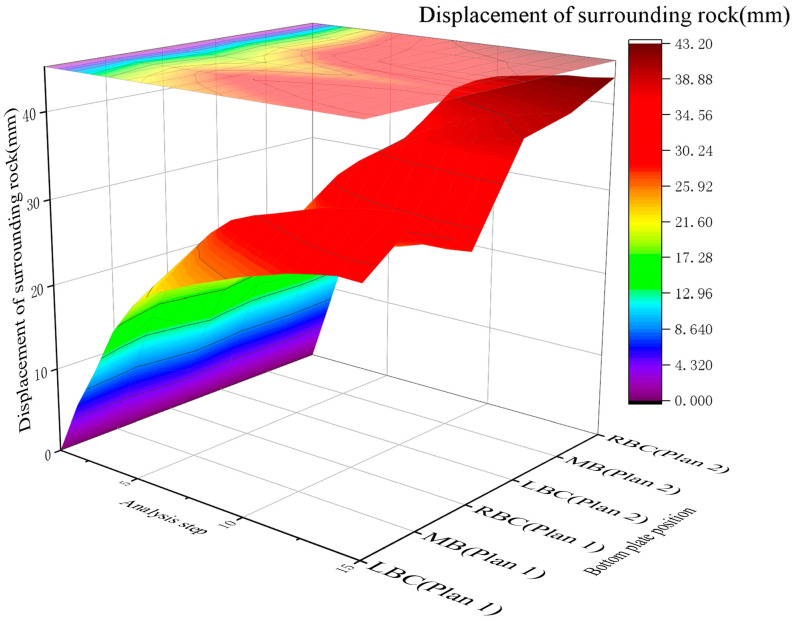
Comparison of the displacement of the surrounding rock in the two schemes.

A comparative analysis of the surrounding rock displacement of Schemes 1 and 2 is shown in Figure 19.

**Figure 20:**
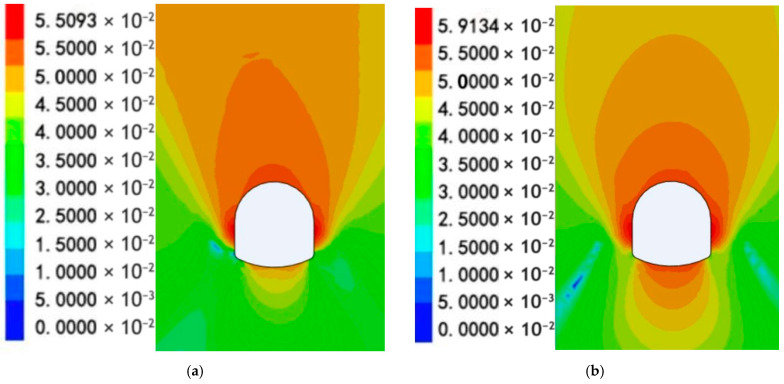
Roadway bottom rock displacement cloud: (**a**) Roadway bottom rock displacement cloud of Scheme 1; (**b**) Roadway bottom rock displacement cloud of Scheme 2.

Figure 20a,b illustrates the displacement of the roadway bottom rock for the two support schemes.

The cloud maps of the displacements in Schemes 1 and 2 are shown in Panels a and b, respectively. Based on the analyses presented in Figure 17, Figure 18, Figure 19 and Figure 20a,b.

The surrounding rock stress release rate averaged 33% for the scheme without RGC (30.2%~30.6% at corners, 38.5% at the middle bottom) and 64% for the scheme with RGC (63.6%~64.0% at corners, 65.5% at the middle bottom). The stress release rate of the scheme with RGC (Scheme 2) was nearly twice that of the scheme without rubber (Scheme 1), increasing from an average of 33% to 64%.

The anchor load statistics showed that Scheme 1 (without rubber) had an average stress of 86.79 MPa (79.22~90.78 MPa), while Scheme 2 (with rubber) had an average stress of 39.69 MPa (38.08~40.87 MPa). Adding the RGC layer significantly reduced the anchor rod load by 57.7% (86.79→39.69 MPa), from 79.22~90.78 MPa to 38.08~40.87 MPa, alleviating the burden on the support structure.

Figure 17 and Figure 20a,b show RGC reduces surrounding rock stress but slightly increases displacement: Scheme 1 (no rubber) has an average bottom slab stress release rate of 33%; Scheme 2 (rubber + ordinary concrete) reaches 64%, releasing 48% total stress and 40% displacement (coordinated stress–displacement adjustment). Postrelease, bottom slab stress stabilizes at 7–9 MPa (lower than initial geostatic stress).

Based on the aforementioned results, the application of a RGC layer between the roadway bottom rock and the ordinary concrete leads to a high stress release rate in the surrounding rock and a reduced load on the support structure.

### 4.3. Influence of Mechanical Parameters of RGC on the Pressure Release Effect

Following the confirmation of the effectiveness of the RGC interlayer, its key mechanical parameters were further evaluated to optimize both stress release and support efficiency in deep high-stress soft rock roadways. The objective was to identify parameters that minimize support loads while maximizing stress release in the bottom rock.

#### 4.3.1. Influence of Elastic Modulus on the Pressure Release Effect

RGC is characterized by relatively low strength and a correspondingly low elastic modulus (*E*), which results in its capacity for large deformation. To understand how the elastic modulus affects pressure release, analyses were conducted on RGC with moduli of 2, 3, 4, 5, and 6 GPa. The findings are detailed below.

1. Surrounding rock stress

**Figure 21:**
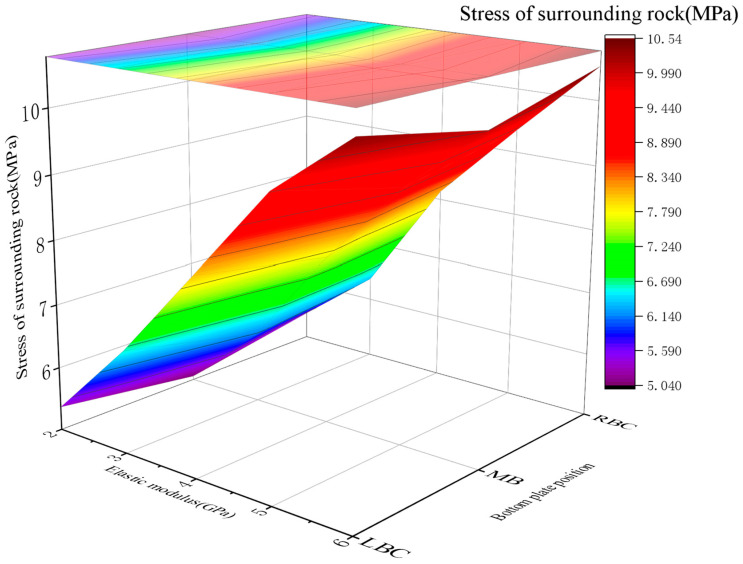
The stress curves of the rock surrounding the roadway bottom with RGC with different elastic moduli.

For RGC with elastic moduli (*E*) of 2 GPa, 3 GPa, 4 GPa, 5 GPa, and 6 GPa, the surrounding rock stress at the roadway bottom showed a gradual increase with *E*: 5.07~5.42 MPa (2 GPa), 6.12~6.43 MPa (3 GPa), 7.64~8.13 MPa (4 GPa), 8.97~9.21 MPa (5 GPa), and 10.02~10.52 MPa (6 GPa). The stress at the right bottom corner was slightly higher than that at other positions across all *E* values (Figure 21).

2. Support load dynamics

**Figure 22:**
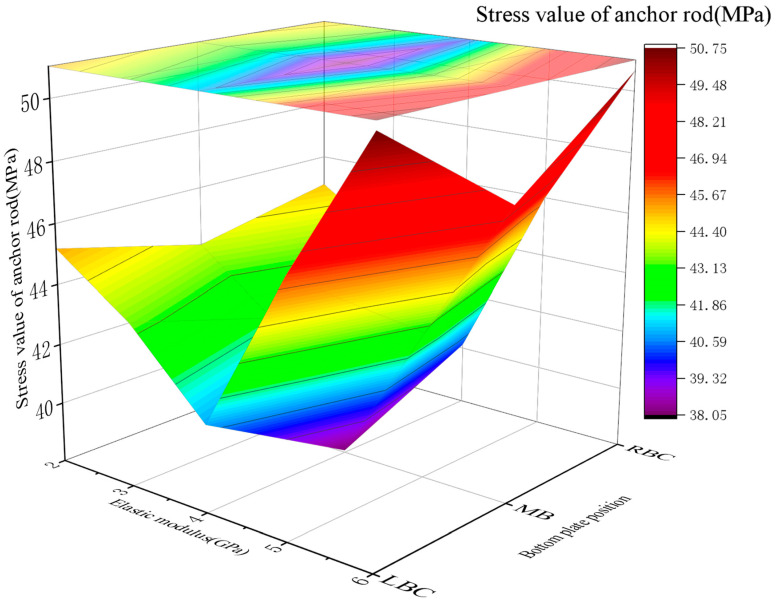
Curve of the stress value of the anchor in RGC with different elastic moduli.

The bolt loads varied with the *E* of RGC: 43.98~45.18 MPa (2 GPa), 42.01~43.42 MPa (3 GPa), 38.08~40.87 MPa (4 GPa), 42.86~46.12 MPa (5 GPa), and 47.49~50.73 MPa (6 GPa). The stress first decreased and then rebounded, with the minimum value observed at *E* = 3~4 GPa (Figure 22).

3. Optimal *E* (3–4 GPa)

This range achieves stress release balance (controlled rock displacement) to prevent bolt overload, with sufficient surrounding rock stress release and no support structure overloading (simulation-validated).

#### 4.3.2. Analysis of the Influence of the UCS on the Pressure Release Effect

To identify the optimal uniaxial compressive strength for maximizing pressure release, simulations were conducted to evaluate the effects of RGC with different UCS values (2, 3, 4, 5, and 6 MPa) on high-stress soft rock roadways.

1. Stress of surrounding rock

With the UCS of RGC increasing from 2 MPa to 6 MPa, the surrounding rock stress at the roadway bottom increased approximately linearly: 5.05~5.26 MPa (2 MPa), 6.52~6.95 MPa (3 MPa), 7.64~8.13 MPa (4 MPa), 9.87~10.26 MPa (5 MPa), and 12.08~12.54 MPa (6 MPa).

**Figure 23:**
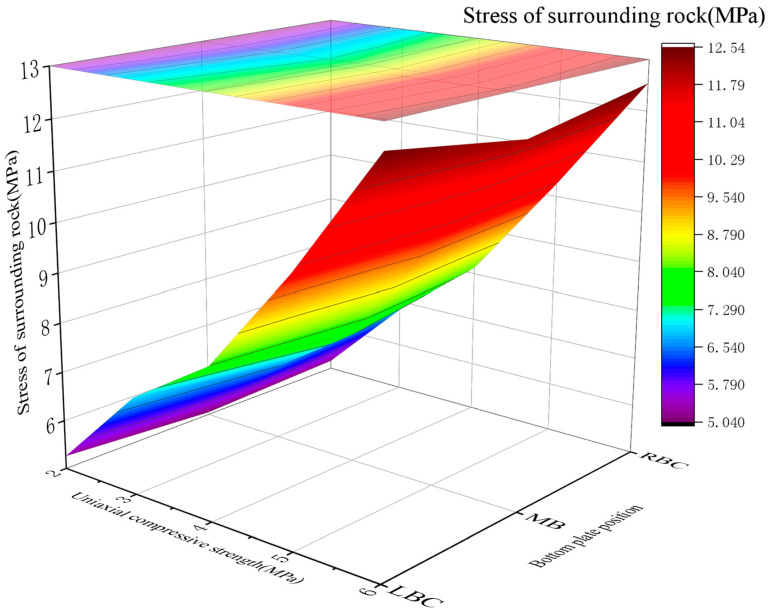
The stress curves of the rock surrounding the roadway bottom under the effects of RGC with different UCSs.

The correlation between the stress in the bottom rock layer and the UCS of the RGC is illustrated in Figure 23. Figure 23 shows high-stress soft rock roadway stress increases approximately linearly with UCS (2–6 MPa).

2. Force on the roadway support structure

**Figure 24:**
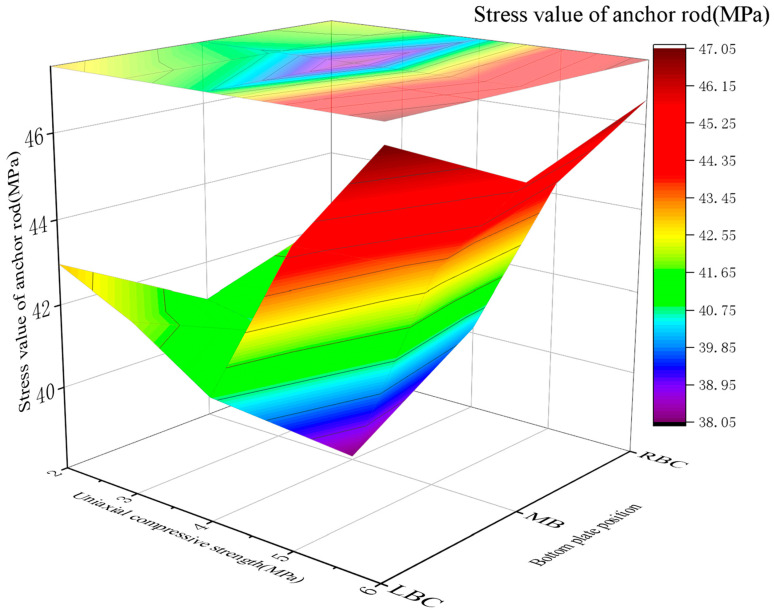
Change in the stress value of the anchor under the effects of RGC with different UCSs.

The bolt stress changed with the UCS of RGC (2~6 MPa): 41.05~42.96 MPa (2 MPa), 40.68~42.08 MPa (3 MPa), 38.08~40.87 MPa (4 MPa), 43.10~44.65 MPa (5 MPa), and 45.48~47.05 MPa (6 MPa). Similar to the *E* trend, the stress first decreased and then increased, with the optimal UCS range of 3~5 MPa. The relationship between the bolt stress and the UCS of the RGC in critical sections of the soft rock roadway is plotted in Figure 24. Figure 24 show bottom plate support bolt load first decreases then increases with UCS (2–6 MPa): lower UCS is not optimal; sufficient UCS increases anchor rod load due to inadequate roadway bottom surrounding rock stress release (similar to *E*’s relationship with bolt stress).

3. Determination of the optimal UCS of RGC

Postsupport surrounding rock stress in soft rock roadways increases with RGC UCS: excessively low UCS causes severe deformation/damage; higher UCS increases rock stress. Anchor rod loads first decrease then rebound (limited rock displacement hinders stress release; incomplete stress transfer increases loads beyond critical UCS). Optimal UCS (3–5 MPa) balances adequate stress release (controlled rock displacement) and anchor rod overload prevention (simulation-validated).

### 4.4. Reasonable Thickness of the Pressure-Releasing Layer Composed of RGC

#### 4.4.1. Determination of the Reasonable Thickness of the Structural Layer

The RGC layer thickness was varied at five levels (200, 250, 300, 350, 400 mm) to analyze its influence on surrounding rock stress and bolt load distribution, with the goal of determining the optimal ordinary concrete layer thickness that minimizes both surrounding rock stress and support structure force.

#### 4.4.2. Analysis of the Influence of RGC Thickness on Roadway Stress

**Figure 25:**
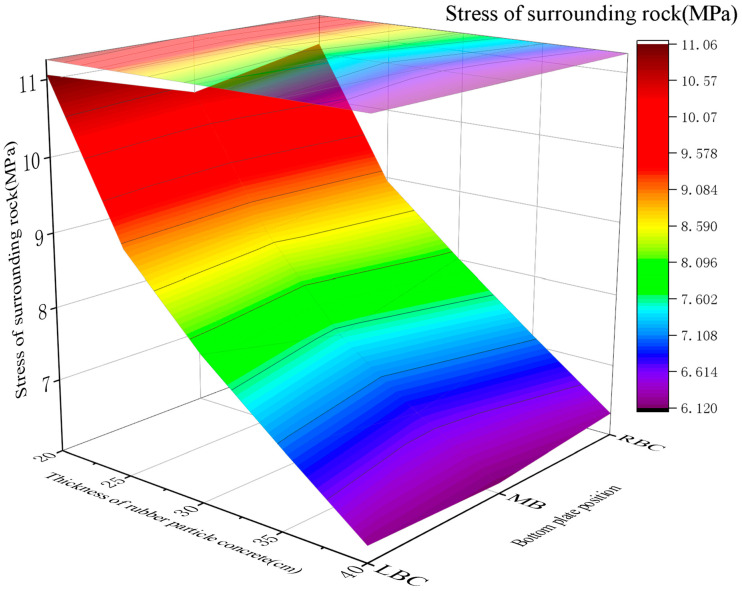
Relationship between RGC thickness and bottom rock stress.

The relationship between the stress at the rear section of the roadway bottom and the RGC layer thickness is shown in Figure 25. As the thickness of the RGC layer increased from 200 mm to 400 mm, the surrounding rock stress at the roadway bottom gradually decreased: 10.47~11.06 MPa (200 mm), 8.72~9.02 MPa (250 mm), 7.53~8.02 MPa (300 mm), 6.75~7.15 MPa (350 mm), and 6.14~6.31 MPa (400 mm). Figure 25 show roadway bottom surrounding rock stress decreases with increasing RGC layer thickness: 200–300 mm thickness yields large stress reduction; 300–400 mm thickness shows slow stress reduction.

#### 4.4.3. Analysis of the Influence of Force on the Anchor Rod Under Different RGC Thicknesses

**Figure 26:**
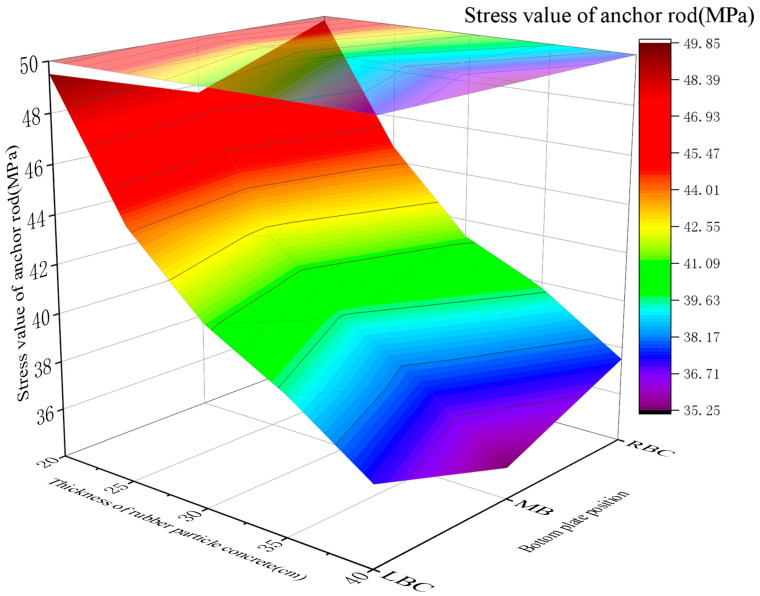
Relationship between the thickness of the RGC and the stress on the anchor rod.

The anchor rod stress decreased with the increasing thickness of the RGC layer (200~400 mm): 47.68~49.83 MPa (200 mm), 42.19~44.65 MPa (250 mm), 39.53~41.29 MPa (300 mm), 37.62~39.76 MPa (350 mm), and 35.27~37.51 MPa (400 mm). The decreasing rate of stress gradually slowed, consistent with existing research findings. Figure 26 show anchor rod load gradually decreases with RGC layer thickness (200–400 mm), with decreasing rate slowing. Existing research [35] corroborates this anchor force behavior. Figure 26 illustrates the relationship between the bolt load and the thickness of the RGC layer.

#### 4.4.4. Determination of the Optimal Thickness of the RGC Layer

Optimal thickness (250–350 mm):

Simulations show rubber granular concrete layer thickness 250–350 mm is optimal: thicker layers reduce surrounding rock stress and anchor rod loads via coordinated deformation; >300–400 mm thickness leads to stress reduction plateau, enlarged excavation area, material waste, and increased instability risk. This range maximizes stress release, minimizes support load, and maintains roadway usability.

In the proposed support system, the RGC layer serves as a flexible pressure-relief component and is combined with conventional anchor-cable support systems. This hybrid design realizes load sharing between the RGC layer (absorbing deformation and releasing stress) and anchor-cables (providing primary structural support), effectively avoiding structural failure caused by the reduction in RGC stiffness under high geostress.

For high rubber content RGC (30–40%), although its uniaxial compressive strength is significantly reduced (to <10 MPa), it exhibits excellent deformability (strain capacity ≥ 0.025) and energy absorption capacity (≥5 kJ/m^3^). This type of RGC is not suitable for primary load-bearing support but can be applied as a passive pressure-relief layer in ultra-high geostress soft rock roadways (geostress > 30 MPa), where deformation control is prioritized over bearing capacity. For conventional high-ground-stress roadways (15–30 MPa), 10–15% rubber content is the optimal range, balancing strength and deformability (UCS ≥ 20 MPa, strain capacity ≥ 0.015). The surrounding rock stress and anchor rod load of the roadway decrease with the increase of RGC layer thickness, and the reduction rate slows down when the thickness exceeds 300 mm. The main reason is that the RGC layer with appropriate thickness can effectively absorb the deformation energy of the surrounding rock and release the ground stress through its own deformation; when the thickness exceeds 300 mm, the stress release effect reaches a plateau because the RGC layer has fully exerted its pressure relief capacity, and the continuous increase of thickness cannot further improve the stress release effect but will cause the waste of materials and the increase of engineering cost.

### 4.5. Progressive Instability and Catastrophic Failure Simulation

To evaluate the performance of RGC under progressive instability and catastrophic failure scenarios, a numerical simulation was conducted to simulate the gradual increase in geostress (from the design stress of 21.5 MPa to 32.25 MPa, 1.5 times the design stress). The results show that the ordinary concrete support system exhibits sudden brittle failure when the geostress reaches 28.6 MPa, while the RGC hybrid support system only shows gradual deformation accumulation without sudden failure when the geostress reaches 32.25 MPa. The RGC layer delays the onset of roadway floor progressive instability by ~40% and reduces the displacement accumulation rate by 52% compared to ordinary concrete.

## 5. Discussion: Application of RGC in Practical Engineering

To verify previous results, a high-stress soft rock roadway in a Hebei deep engineering project was selected. Finite element simulations studied different schemes based on bottom plate pressure release principles and parameters; the optimal scheme was applied on-site with 60-day deformation monitoring to verify feasibility.

### 5.1. Support Scheme and Supporting Parameters

Support scheme:

Based on Hebei’s deep engineering actual situation and above analysis, the main support scheme is full-section bolt, anchor cable, grouting, shotcrete and metal mesh, with secondary support at 28–29 days post-initial support and bottom plate reinforcement. Two schemes are proposed via engineering analogy (Schemes 3 and 4):

Scheme 3:

Full-section anchor bolt support + anchor cable + grouting + shotcrete and hanging metal mesh primary support. Reinforced concrete laying crown for secondary support. The bottom plate bolt, T-shaped steel belt, and anchor cable harness are used to reinforce the bottom plate.

Scheme 4:

A 150 mm ordinary concrete layer is sprayed on the bottom plate to create a 350 mm RGC layer, followed by a 115 mm ordinary concrete layer. The other components are the same as those in Scheme 3.

Support Parameters:

Roof/Rib bolts: high-strength prestressed resin bolts with a diameter of 22 mm and a length of 2400 mm were used, installed at a spacing of 700 mm.

Grouting bolts: seamless grouting bolts (Ø20 × 2200 mm; spacing of 1400 mm) were alternated with resin bolts every other row.

Anchor cables: high-strength cables (Ø22 × 10 m; spacing of 1400 mm) for roofs/ribs.

Shotcrete: C20 concrete layer (150 mm thick).

Metal mesh: Ø6.5 mm mesh (100 × 100 mm grid).

Steel ladders: Ø20 mm reinforcing ladders linked to resin bolts.

T-Steel belts: GDII140/20 mm specifications.

U36 steel supports: inverted arch U36 supports (0.5 m spacing) within 3 m of the gate openings.

Grouting: cement slurry (1.5→2.5 MPa pressure).

Bottom bolts: high-strength bolts (Ø22 × 2400 mm; 700 mm spacing) with 140 mm T-steel strips.

Bottom grouting bolts: as above (1400 mm spacing).

Bottom cable bundles: 3 × Ø22 × 10 m steel strands (2800 mm spacing; No.11 I-beams between bundles) within 20 m of the gate openings.

**Figure 27:**
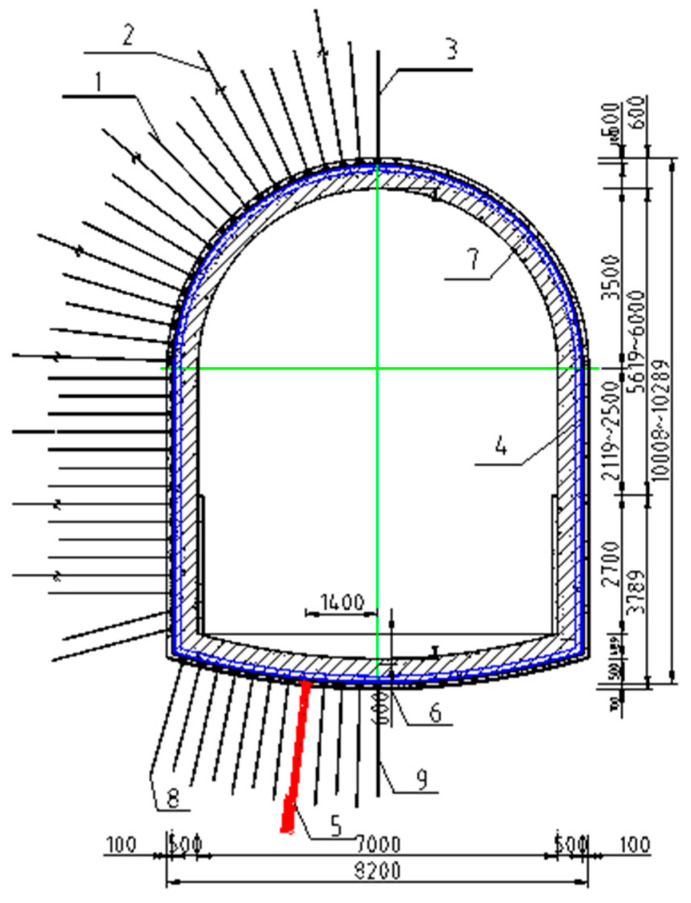
Support structure of Cross-Section 1-1 of the ingate roadway.

**Figure 28:**
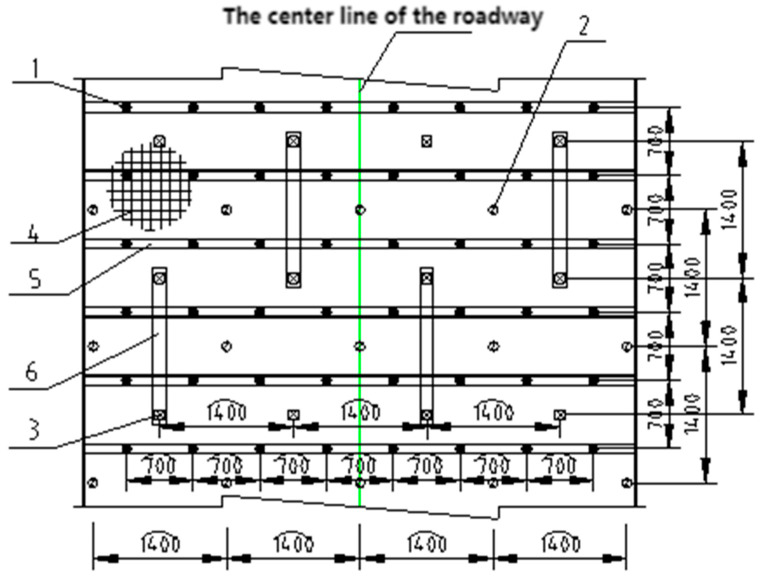
Arrangement of the anchor and bolt mesh.

Layout: support structure (Figure 27); bolt/cable arrangement (Figure 28).

From Figure 27: 1-High-strength bolts for roof and rib; 2-anchor cable; 3-grouting bolts for roof and rib; 4-U-steel; 5-bottom anchor cable bundles; 6-reinforcing steel ladder; 7-secondary lining; 8-high-strength bolts for the bottom; 9-bottom grouting bolts.

From Figure 28: 1-High-strength prestressed anchor bolt; 2-grouted bolt; 3-high-strength anchor cable; 4-metal mesh; 5-reinforcing steel ladder beam; 6-T-shaped steel belt.

### 5.2. Numerical Simulation and Analysis of Different Support Schemes

**Figure 29:**
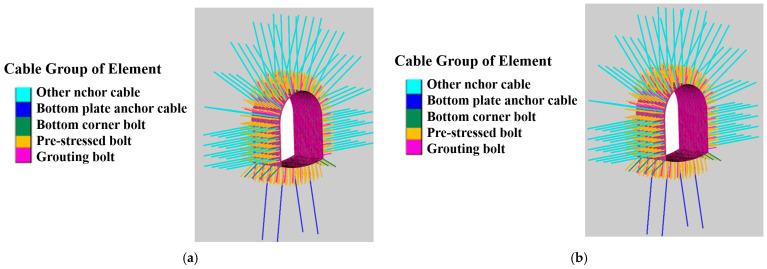
Numerical simulation diagram: (**a**) Numerical simulation diagram of Scheme 3; (**b**) Numerical simulation diagram of Scheme 4.

FLAC simulations were conducted for Schemes 3 and 4 (hexahedral elements, same meshing as above; Coulomb–Mohr criterion, structural damping factor 0.03) to analyze minimum principal stress, support structure stress, and roadway displacement (Figure 29a,b). The optimal scheme was applied on-site to verify reliability.

The core conclusions are based on full 3D numerical simulations (ABAQUS/FLAC) that incorporate realistic geological discontinuities and in-situ stress conditions, which were validated against field data. We note that future work will use discrete element method (DEM) models to explicitly represent rock joints and anisotropic behavior for more comprehensive analysis.

#### 5.2.1. Minimum Main Stress Analysis

**Figure 30:**
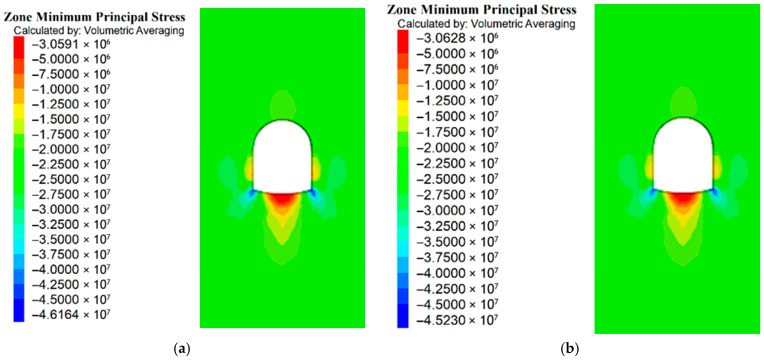
Minimum principal stress: (**a**) Minimum principal stress of Scheme 3; (**b**) Minimum principal stress of Scheme 4.

For the two support schemes, the minimum principal stress of the roadway bottom was lower in Scheme 4 (with RGC) than in Scheme 3 (without rubber): Scheme 3 had 40.2~42.6 MPa (40.2 MPa at the middle bottom, 42.5~42.6 MPa at the left/right bottom corners), while Scheme 4 had 36.8~38.1 MPa (36.8 MPa at the middle bottom, 37.8~38.1 MPa at the left/right bottom corners), which reduces stress concentration and facilitates sufficient stress release (Figure 30a,b). As shown in Figure 30, Scheme 4 exhibits a lower minimum principal stress than Scheme 3, thus alleviating floor stress concentration and achieving more adequate surrounding rock stress release. RGC is feasible for roadway ground stress release.

#### 5.2.2. Force Analysis of the Bottom Plate Support Structure

**Figure 31:**
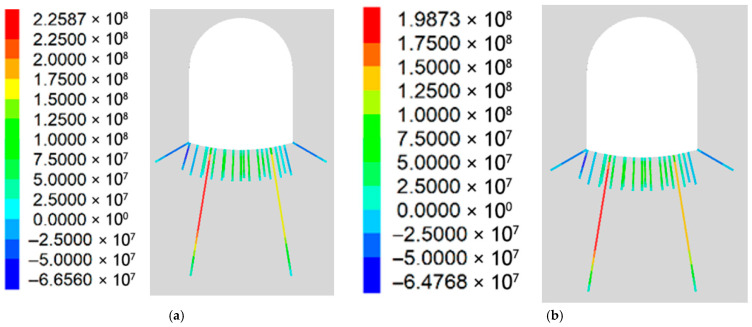
Stress acting of bottom plate support structure: (**a**) Scheme 3; (**b**) Scheme 4.

Because Scheme 4 includes a RGC layer at the bottom plate, to reflect the results more clearly and conveniently, the support structure of the bottom plate is selected for detailed analysis. Figure 31a,b shows RGC improves bottom plate support structure stress: maximum load decreases from 226 MPa to 198 MPa vs. Scheme 3, reducing support structure load and bearing capacity loss risk.

The stress relief and deformation control performance of RGC was further compared with two mainstream roadway pressure-relief technologies (foam concrete backfilling and independent anchor-cable support) based on published data from similar high-ground-stress soft rock roadway projects. The results show that RGC achieves a 64% stress release rate, which is slightly lower than foam concrete (≈68%) but 3.2 times that of independent anchor-cable support (≈20%). In terms of economic efficiency, RGC has a 25% lower material cost than foam concrete and a 40% lower long-term maintenance cost than anchor-cable support.

In addition, RGC has unique engineering advantages: it is compatible with conventional shotcrete construction processes (no additional equipment required) and realizes waste valorization, which makes it a more sustainable alternative compared to other pressure-relief technologies.

#### 5.2.3. Rock Displacement Distribution Around the Roadway

**Figure 32:**
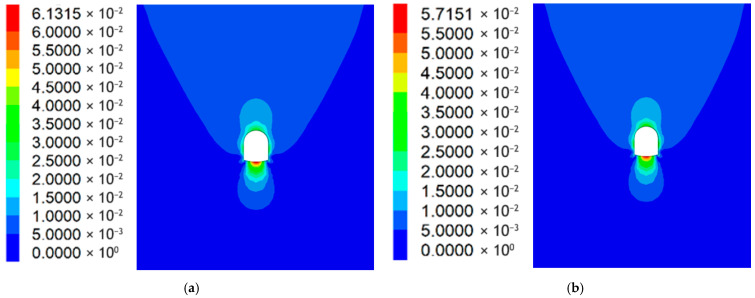
Surrounding rock displacement contour map: (**a**) Scheme 3; (**b**) Scheme 4.

Figure 32a,b shows Scheme 4’s bottom plate displacement (57 mm) is slightly lower than Scheme 3’s (61 mm), attributed to surrounding rock displacement during pressure release. RGC enables full surrounding rock stress release, avoids excessive support structure stress and bearing capacity loss, and stabilizes surrounding rock displacement. Thus, Scheme 4 is optimal.

#### 5.2.4. Application Effect of the Project Site

**Figure 33:**
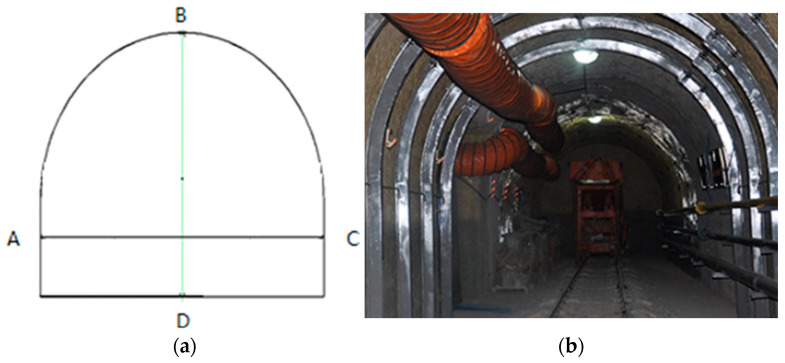
Layout diagram of the measuring site: (**a**) Schematic of the measurement points; (**b**) Photograph of the deep engineering roadway project.

The optimal support scheme (Scheme 4) is applied at the engineering site, and the deformation of the engineering site is monitored. Figure 33a,b shows the layout diagram of the measuring site.

Monitoring settings: Device-Guanglu 321-134W high-range electromechanical micrometer; frequency—once/24 h, 60 days (extended if unstable); measurement points—cross-shaped layout (Figure 33a,b), DTB (B–D line, top/bottom plate deformation), DTS (A–C line deformation); three measuring stations (10 m spacing: Station 1–3).

Equipment Installation and Labeling: Install the measuring piles between point B and point D of Station one. Place the monitoring equipment on the surface of the measuring piles. This equipment is labeled as No. 1. The recorded data will be Station one’s DTB and is labeled as DTB_1_. Proceed in the same manner. Station one’s DTS is labeled as DTS_2_. Station two’s DTB is labeled as DTB_3_. Station two’s DTS is labeled as DTS_4_. Station three’s DTB is labeled as DTB_5_. Station three’s DTS is labeled as DTS_6_.

Calibration: Equipment calibrated in the laboratory pre-use (standard blocks, 20 ± 1 °C) to ensure linear error < ±0.5% of full scale; on-site checks every 15 days with standard gauges to ensure data reliability.

**Figure 34:**
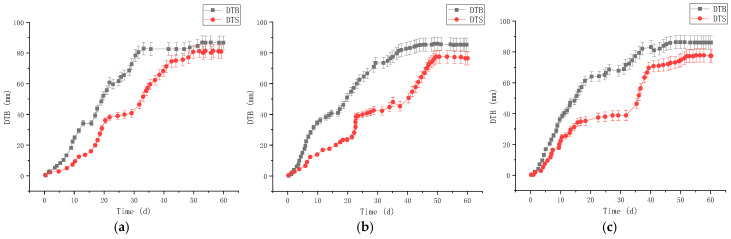
Application at the engineering site (before support work): (**a**) Station one (DTB_1_ and DTS_2_); (**b**) Station two (DTB_3_ and DTS_4_); (**c**) Station three (DTB_5_ and DTS_6_).

**Figure 35:**
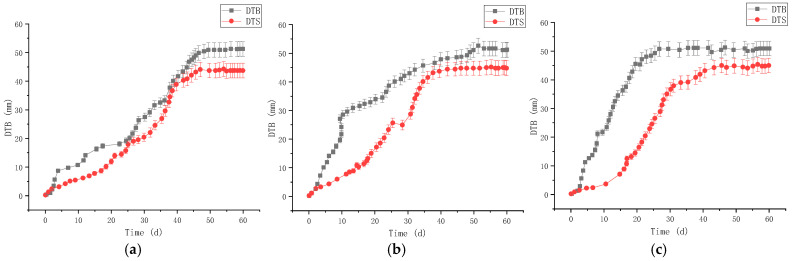
Application at the engineering site (after support work): (**a**) Station one (DTB_1_ and DTS_2_); (**b**) Station two (DTB_3_ and DTS_4_); (**c**) Station three (DTB_5_ and DTS_6_).

The corresponding monitoring results are presented in Figure 34 and Figure 35.

The field test site is a deep high-ground-stress soft rock roadway in a coal mine in Hebei Province, China, with a buried depth of 800 m and an in-situ vertical stress of 21.52 MPa (data source: in-situ stress measurement by the mine’s geotechnical engineering department using the stress relief method). The surrounding rock of the roadway is mainly siltstone and mudstone, with its mechanical parameters obtained via laboratory triaxial compression tests of in-situ rock samples (in accordance with ISO 17892-8:2020) in the Shandong Provincial Key Laboratory of Civil Engineering Disaster Prevention and Mitigation. The field deformation monitoring data were collected using a Guanglu 321-134W high-range electromechanical micrometer (calibrated by the National Metrology Institute of China), with a monitoring frequency of once per 24 h for 60 consecutive days; the measurement points were arranged in a cross-shaped layout at three measuring stations (10 m spacing), and the test method followed the Chinese National Standard GB 50026-2020 (Code for Geotechnical Investigation and Surveying) and ASTM D4729-21 (Standard Test Method for Measuring Horizontal and Vertical Displacements in Soil and Rock Using In-Place Extensometers). The key site data include roadway width (5 m), height (4.5 m), surrounding rock uniaxial compressive strength (5–10 MPa), and initial floor heave deformation (86.37 mm before support).

During the field construction of RGC shotcrete, a continuous mixing process with a twin-shaft mixer was adopted to ensure the uniformity of rubber particle distribution. After the construction of the RGC layer, 12 core samples (φ50 mm × 100 mm) were taken from different positions of the roadway bottom. The rubber content of each core sample was tested, and the coefficient of variation (CV) was <9.5%, indicating good mixing uniformity of RGC in field engineering. The uniaxial compressive strength of field core samples was 19.2–21.5 MPa, which is consistent with the laboratory test results (≥20 MPa), verifying the consistency of RGC mechanical properties between laboratory and field.

Figure 34 and Figure 35 shows: Pre-support—DTB stabilized at 86.37 mm (average of 3 stations) after 50 d, DTS at ~78.87 mm (slightly lower); post-support—DTB/DTS stabilized at 51.74 mm/44.91 mm (average), with effective deformation control meeting engineering requirements.

This study’s optimal support scheme effectively controls roadway bottom drum via surrounding rock deformation control, validating RGC’s engineering applicability. Results provide a reliable waste rubber treatment concept and contribute to environmental protection.

Rubber particles’ viscoelastic properties enhance concrete’s impact energy absorption but may cause fatigue degradation during long-term service: 50 freeze–thaw cycles reduce the elastic modulus of 20% rubber samples by 18% (microcrack expansion at rubber–mortar interface, rubber molecular chain breakage). Recommendations: Quantify fatigue life via three-point bending tests (rubber concrete fatigue limit ~60–75% of ordinary concrete under typical load spectra); surface silane impregnation or nano-silicon dioxide addition delays aging in dynamic loading environments (e.g., road bases), extending service life by >40%. Future research should establish a quantitative model between rubber content, particle size distribution, and fatigue performance.

It is noted that future work will include long-term monitoring (≥12 months) at the same site to quantify creep behavior and progressive deformation, as well as accelerated laboratory aging tests to simulate a 20-year service life of RGC.

It should be noted that the current field validation was conducted in a typical soft rock formation (siltstone and mudstone) under high geostress conditions. Therefore, the conclusions drawn in this study are most applicable to similar geological and engineering backgrounds, and caution should be exercised when extending these results to hard rock or low-stress environments.

Future field trials are planned to be carried out at two additional sites with distinct geological characteristics, including sandstone and limestone formations with varying groundwater conditions. These extended tests aim to quantify the performance variability of RGC under different scenarios and further validate its broad applicability in geotechnical engineering.

For special engineering scenarios with high dynamic loads (e.g., blasting zones) or ultra-high geostress, the mechanical performance of RGC can be further optimized by hybrid modification with steel fibers (1–2 vol.%) or polymer modifiers (e.g., epoxy resin). Relevant studies have verified that this modification method can improve the UCS of RGC by 25–30% without reducing its energy absorption capacity and deformability.

#### 5.2.5. Scientific Implications of the Findings

The scientific implications of this study’s findings are mainly reflected in two aspects: (1) sustainable material science: this study realizes the high-value utilization of waste tire rubber in geotechnical engineering, providing a new technical route for the sustainable management of solid waste and the development of low-intensity high-toughness geotechnical materials; the established RGC parameter optimization system clarifies the quantitative relationship between the composition and performance of rubberized concrete for pressure relief, enriching the theoretical research on modified concrete materials. (2) geotechnical engineering mechanics: the proposed RGC pressure-relief layer mechanism reveals the new law of “flexible material deformation to release surrounding rock stress” in high-ground-stress soft rock roadways, breaking the limitation of conventional “rigid support to resist ground stress” design concept; the hybrid support system of RGC +anchor-cable provides a new mechanical model for the stability control of deep soft rock roadways, which is of great significance for the development of deep geotechnical engineering support technology.

### 5.3. Environmental and Economic Benefits

A preliminary cradle-to-gate lifecycle assessment (LCA) was conducted to quantify the environmental benefits of RGC, in accordance with the LCA framework for rubberized concrete specified in ISO 14040/14044. The results show that replacing 10–15% of concrete aggregates with waste tire rubber reduces the carbon footprint of the shotcrete by ~22% compared to ordinary concrete, mainly due to the reduction in cement consumption and the avoidance of waste tire landfill/incineration emissions (≈0.18 t CO_2_-eq per ton of RGC).

From an economic perspective, the field application in Hebei shows that RGC reduces the total roadway support cost by ~18%: the material cost is reduced by 15% due to the use of waste rubber, and the maintenance cost is reduced by ~42% by mitigating floor heave deformation (avoiding repeated repair and reinforcement). A full cradle-to-grave LCA including the end-of-life phase of RGC will be the focus of subsequent research.

Contributions to Existing Knowledge: this study makes three key contributions to the existing knowledge in the fields of sustainable geotechnical materials and deep roadway engineering: (1) filling the research gap: the study for the first time systematically investigates the pressure relief mechanism and field application of RGC in high-ground-stress soft rock roadway floor heave control, supplementing the lack of research on RGC in high-stress geotechnical engineering scenarios in existing studies; (2) proposing new methods: a complete research method of “laboratory tests-theoretical derivation-numerical simulation-field validation” for RGC application in geotechnical engineering is established, providing a standardized research framework for the study of sustainable geotechnical materials; (3) providing engineering references: the optimized RGC parameter system and hybrid support design scheme proposed in this study provide direct technical references for the engineering practice of high-ground-stress soft rock roadway floor heave control, and the field validation results verify the feasibility and effectiveness of the technology, which has important guiding significance for similar engineering projects.

## 6. Conclusions

This study innovatively applies waste tire-derived RGC to the field control of high-ground-stress soft rock roadway floor heave, which is the primary novelty of this work compared with existing rubberized concrete studies (mostly laboratory/numerical research). The field application verifies the engineering feasibility of RGC for geotechnical stress relief, and the optimized RGC parameters (10–15% rubber content, 250–350 mm thickness) and hybrid support design provide a new sustainable solution for deep roadway stability control, realizing the dual goals of waste tire valorization and geotechnical engineering safety. RGC was used as a pressure release layer; its mechanical characteristics and parameters, as well as layer thickness, were optimized via laboratory tests and theoretical deductions. The optimal support scheme was obtained, applied to a Hebei deep engineering site, and validated via field monitoring (effective surrounding rock deformation control and bottom drum improvement). Without RGC, the average bottom slab surrounding rock stress release rate is 33%; with RGC between surrounding rock and ordinary concrete, it reaches 64%, effectively alleviating bottom slab surrounding rock pressure. Results provide new waste rubber disposal ideas: this approach protects the environment, reduces waste tire treatment and bottom plate support material costs, and is innovative for high-stress soft rock roadway stability reinforcement. The conclusions are as follows:

1. The pressure-release mechanism mitigates surrounding rock stress, thereby enhancing support safety and economic efficiency.
2. Numerical simulations demonstrate that placing an RGC layer between ordinary concrete and the surrounding rock achieves an optimal pressure release effect, and the relevant mechanical parameters and optimal layer thickness of RGC were determined.
3. A numerical model was established based on a deep engineering project in Hebei; the optimal support scheme was designed, field-applied, and validated to effectively control the deformation of the floor surrounding rock.
4. The incorporation of waste rubber into concrete yields significant environmental benefits, as it reuses waste rubber, waste tires and other rubber products as particles added to the concrete. This technique reduces the accumulation of rubber waste and reduces environmental pollution. Rubber can also replace some traditional materials in construction engineering, thereby saving energy and reducing consumption. This method not only reduces the economic cost of dealing with discarded tires but also reduces the cost of materials used in bottom plate support and helps the resource cycle and sustainable development. Furthermore, this research outcome also holds potential application value in specific scenarios of low intensity and high toughness. However, its durability and other characteristics still need to be further improved.

In conclusion, this study successfully demonstrates a closed-loop strategy for waste tires, repurposing them into a critical component of geotechnical support systems. The developed RGC, functioning as a stress-release layer, has proven effective in mitigating floor heave in demanding field conditions. More than an engineering success, this work provides a replicable technological case study for achieving waste diversion and resource recovery. It underscores the significant potential of coupling waste valorization with infrastructure development, offering a dual- benefit solution that aligns with the principles of a circular economy and advances sustainable waste management practices.

## Data Availability

The original contributions presented in this study are included in the article. Further inquiries can be directed to the corresponding author.
